# Co-Evolutionary Sensor Deployment Optimization for Layered Low-Altitude Airspace Monitoring Under Worst-Case Traffic Composition

**DOI:** 10.3390/s26216737

**Published:** 2026-10-24

**Authors:** Xianrong Yan, Tongbo Wang, Jihong Zhu

**Affiliations:** Department of Precision Instrument, Tsinghua University, Beijing 100084, China; yanxr22@mails.tsinghua.edu.cn (X.Y.); jhzhu@tsinghua.edu.cn (J.Z.)

**Keywords:** low-altitude airspace monitoring, early-warning sensor network, sensor deployment optimization, competitive co-evolution, worst-case robust optimization, heterogeneous low-altitude targets, many-objective optimization

## Abstract

Layered sensor networks that monitor low-altitude airspace must detect heterogeneous aerial traffic, yet sensor deployment is usually optimized against a single fixed traffic case and can leave an entire target kind undetected when the composition changes. This paper formulates deployment against worst-case traffic composition through a high–low transition (HL) trajectory model, a library of 35 target templates in four kinds, and a per-kind fairness objective $f_{4}$, the worst per-kind miss rate. An adaptive co-evolution (ACE) framework trains deployments against a co-evolved, kind-diverse population of traffic cases with a hall of fame and a robust final selection, and ACE-A6 instantiates it with a sensor-count dispatcher. Over four scenarios with five, four, three, and two sensors; 30 seeds; and two independent sets of 60 held-out cases, ACE-A6 lowers the worst per-kind miss rate of four state-of-the-art evolutionary optimizers on the four-, three- and two-sensor scenarios with large effect sizes (Cliff’s δ≥ 0.44, Holm-corrected p<0.05). Equal-budget experiments locate the gain in the training framework: each of five optimizers trained inside ACE breaks the $f_{4,\max}=1$ ceiling, and ACE-A6 performs on par with them. The advantage persists under probabilistic detection, masking, position error and moderate library shift.

## 1. Introduction

Layered sensor networks that monitor the airspace around a fixed site no longer face homogeneous, high-altitude traffic. They must detect a heterogeneous mix of high-altitude targets (T1, T2), low-altitude targets with a high–low transition profile (T3) and surface-skimming targets (T4) [1,2]. The same shift is visible in civilian low-altitude airspace, where small unmanned aircraft are hard to detect with a single sensor type [3] and distributed ground-based sensing networks are being tested for advanced air mobility [4]. Prior optimization frameworks formulate sensor deployment as a multi-objective problem against a single fixed traffic case [5,6]. A deployment tuned to that case can still fail on a different composition drawn from the same set of target templates, and such a failure tends to concentrate on one target kind. Throughout this paper, lengths and altitudes are expressed in normalized simulation units (n.u.); Section 3.4 lists the complete parameter set needed to reproduce every result and states the scope of the sensing model.

Two things are therefore missing from the standard formulation, namely, a target model in which low-altitude kinds are distinguishable from high-altitude ones, and an objective that notices when one kind is missed entirely. Both are supplied here. A library of 35 target templates in four kinds replaces the fixed scenario, and its low-altitude members follow an explicit high–low transition (HL) trajectory. A per-kind fairness term joins the aggregate objectives, and the deployment is trained against a population of traffic cases composed from that library. For this formulation, this paper proposes ACE (adaptive co-evolution), a training framework with three components. A population of traffic cases co-evolves with the deployment population, a clustered hall of fame retains the hardest cases found so far, and a robust final selection re-evaluates candidates on the accumulated case set. ACE-A6 instantiates the framework with a dispatcher that selects one of four internal modes according to the sensor count $N_{s}$.

ACE-A6 is benchmarked against eight external methods (Random Search, the non-dominated sorting genetic algorithm II (NSGA-II), its priority-weighted variant PW-NSGA-II and its reference-point successor NSGA-III, and the state-of-the-art evolutionary algorithms coevolutionary constrained multi-objective optimization (CCMO), the constrained two-archive evolutionary algorithm (C-TAEA), the many-objective evolutionary algorithm with Pareto front density estimation (MaOEA-PDE) and the enhanced multi-objective Genghis Khan shark optimizer (EnMOGKSO)) on four deployment scenarios with five, four, three and two sensors (S1–S4); 30 random seeds; and two independent sets of 60 held-out traffic cases. Dedicated experiments answer four further questions. The first asks whether the improvement comes from the optimizer or from the training framework and places five external optimizers inside ACE at an equal evaluation budget. The second separates the contributions of the fairness objective and the training cases. The third tests the sensor-count dispatch rule on ten scenarios outside its design set and the fourth re-evaluates the deployments under a non-ideal sensing model and shifts of the target library.

The main contributions of this paper are the following.

- **A heterogeneous target library and an HL-profile kinematic model.** The library holds 35 templates spanning four kinds. Modeling the high–low transition explicitly exposes the collapse of the line-of-sight (LoS) horizon of a low-mounted sensor from 390 n.u. to 104 n.u. when a target descends from 8 n.u. to 0.4 n.u.
- **A per-kind fairness objective** $f_{4}=\max_{k}{\mathrm{MR}}_{k}$, the worst per-kind miss rate, which exposes failures that the aggregate miss rate hides. A factorial experiment shows that $f_{4}$ acts through the training cases. It has no effect under single-case training and lowers the worst per-kind miss rate when the deployment is trained on several kind-diverse cases.
- **The ACE training framework and its instance ACE-A6.** Against the four state-of-the-art optimizers in their published single-case form, ACE-A6 lowers the worst per-kind miss rate on the four-, three- and two-sensor scenarios with large effect sizes. An independent test set confirms the result. The framework is not tied to one optimizer. Each of five external optimizers placed inside ACE also breaks the $f_{4,\max}=1$ ceiling, and ACE-A6 performs on par with them at an equal evaluation budget.
- **A stress-tested evaluation.** Measured evaluation budgets, same-framework baselines, ten held-out scenarios, a stochastic sensing model with five non-ideal effects, and eight target-library shifts delimit where the conclusions hold.

The remainder of this paper is organized as follows. Section 2 reviews related work. Section 3 provides a background on sensing physics, HL-profile kinematics and the parameter set. Section 4 formulates the heterogeneous-target deployment problem. Section 5 presents the ACE framework and ACE-A6. Section 6 reports the experiments. Section 7 discusses mechanism and limitations and Section 8 concludes.

## 2. Related Work

**Sensor deployment optimization.** Wireless sensor network deployment has been extensively studied [5,7], and evolutionary formulations have been applied to drone-swarm deployment for coverage maximization [8]. Recent work formulates 3D deployment as constrained multi-objective optimization [6,9,10], considering coverage, connectivity, and cost; terrain-aware deployment replaces the disc model by a propagation model evaluated over 3D terrain [10]. Zou and Chakrabarty [11] bring probabilistic sensing models, in which the detection probability decays with range instead of switching off at a fixed radius, into deployment optimization. Network-configuration design for coherent multistatic sensing has been studied from a geometry-optimization perspective [12], while robust formulations explicitly hedge against worst-case conditions [13,14,15]. Resource management and configuration optimization for distributed sensing networks has been actively studied in recent aerospace systems research [16,17]. A related body of literature studies layered early-warning architectures, balanced-coverage placement of small sensor nodes, and detection and tracking systems for small unmanned aircraft [18,19]. The present work differs in two aspects. First, heterogeneous *targets* are modeled with an explicit HL-profile trajectory, rather than heterogeneous *sensors*. Second, the deployment is trained against a population of hard traffic compositions rather than a single nominal one.

**Many-objective evolutionary optimization.** NSGA-II [20] established the elitist non-dominated sorting paradigm, and recent surveys consolidate the modern many-objective landscape [21]. NSGA-III [22] extends NSGA-II to many objectives via reference points; reference-vector guided methods and their self-adaptive successors [23] are notable variants. Recent extensions include adaptive reference vectors based on Pareto density (MaOEA-PDE [24]), dynamic-penalty constrained multi-objective evolutionary algorithms [25], surrogate-assisted reference-vector updates [26,27] and constrained many-objective formulations tailored to unmanned aerial vehicle (UAV) mission planning [28]. Constrained variants C-TAEA [29] and CCMO [30] maintain dual archives or dual populations for convergence and feasibility, and the angle-based constrained dominance principle of [31] is a further variant. The PlatEMO platform [32] collects reference implementations of these algorithms; the baselines in this paper are Python re-implementations that follow the published designs (Section 6.1). Beyond benchmark suites, multi-objective optimization has been applied to diverse aerospace tasks, including cooperative mission planning for multiple spacecraft [33] and development-assurance-level assignment in aircraft and system development [34].

**Worst-case and co-evolutionary optimization.** Competitive co-evolutionary algorithms model worst-case conditions with two interacting populations [35,36] and are related to generative modeling [37], bilevel optimization [38] and black-box min–max optimization [39]. Recent applications span co-evolutionary computation for deep learning [40], many-objective co-evolution for multi-UAV coalition formation [41] and security games [42]. Game-theoretic resource allocation under worst-case conditions has likewise been studied in the security games literature [43]. The ACE framework adapts this paradigm to discrete case composition (selecting 12 templates from a library of 35) coupled with continuous deployment (sensor positions in $R^{D}$).

## 3. Background

### 3.1. Network Architecture and Detection Physics

**Network architecture.** A layered network protecting a fixed site maintains three kinds of sensor: a low-mounted surface platform (Sensor-A), a high-altitude airborne platform (Sensor-B) and a mid-altitude airborne platform (Sensor-C). Sensor-A operates at surface level ($h_{s}\approx 0.028$ n.u. mounting height) with limited range. Sensor-B operates at $h_{s}=8$ n.u. with extended range. Sensor-C operates at $h_{s}=5$ n.u. with intermediate range. The deployment problem positions these sensors in the 2D horizontal plane, with a fixed altitude per sensor type, to optimize detection of incoming targets.

**Detection physics.** Two physical constraints limit the effective detection radius $R_{\mathrm{eff}}$ of a sensor against a target:

**(1) Line-of-Sight (LoS) Horizon.** Surface curvature limits direct line-of-sight detection. Under the standard refractive propagation model,

(1) $$R_{\mathrm{hor}}\left(h_{s},h_{t}\right)=\kappa \left(\sqrt{h_{s}}+\sqrt{h_{t}}\right),\;\kappa =130.3,$$

where κ is the normalized propagation constant and all altitudes are in n.u.

**(2) Sensing-Range Model.** For a target with cross section (RCS) $\sigma_{t}$, the detection range scales as the fourth root of the cross-section ratio:

(2) $$R_{\mathrm{RCS}}\left(\sigma_{t}\right)=R_{\mathrm{ref}}{\left(\sigma_{t}/\sigma_{\mathrm{ref}}\right)}^{1/4},$$

where $R_{\mathrm{ref}}$ is the reference range at $\sigma_{\mathrm{ref}}$. The effective detection range is the minimum of the two:

(3) $$R_{\mathrm{eff}}\left(h_{s},h_{t},\sigma_{t}\right)=\min\left(R_{\mathrm{hor}}\left(h_{s},h_{t}\right),\;R_{\mathrm{RCS}}\left(\sigma_{t}\right)\right).$$

A target is confirmed once it lies inside the effective radius of at least one sensor for three consecutive time steps.

### 3.2. Low-Altitude Targets and Trajectory Profiles

**The low-altitude target challenge.** Low-altitude targets exploit surface curvature by descending en route. Table 1 computes $R_{\mathrm{hor}}$ for the three sensor types against high-altitude (8 n.u.) and low-altitude (0.4 n.u.) targets.

The −73% horizon collapse for Sensor-A against a target at $h_{t}=0.4$ n.u. is the physical motivation for the heterogeneous target library: a homogeneous high-altitude traffic model overstates the horizon of the low-mounted sensor by a factor of 3.7.

**High–low transition (HL) profile.** A typical HL-profile target enters at high altitude ($h_{\mathrm{high}}=8$ n.u.), cruises until reaching radial distance $r_{\mathrm{dive}}$ from the site and then descends linearly to surface-skimming altitude $h_{\mathrm{low}}\in \left[0.05,0.4\right]$ n.u. by reaching $r_{\mathrm{skim}}$. The altitude profile is parameterized as a piecewise function of radial distance:

(4) $$h\left(r\right)=\left\{\begin{array}{cc} h_{\mathrm{high}} & r\geq r_{\mathrm{dive}}\\ h_{\mathrm{high}}+\left(h_{\mathrm{low}}-h_{\mathrm{high}}\right)\frac{r_{\mathrm{dive}}-r}{r_{\mathrm{dive}}-r_{\mathrm{skim}}} & r_{\mathrm{skim}}<r<r_{\mathrm{dive}}\\ h_{\mathrm{low}} & r\leq r_{\mathrm{skim}} \end{array}\right.$$

The horizontal motion is a straight-line approach to the site at constant speed *v*, parameterized by the initial angle $\theta \in [-65^{\circ },65^{\circ }]$ from the principal axis of the site. The model captures both segments of the flight. During the high-altitude cruise, detection is mainly limited by the cross section, and during the low-altitude segment, detection by the low-mounted sensor is limited by the horizon.

**Worst-case traffic composition.** A deployment can be judged against a single fixed case or against the hardest case that can be composed from a library M of target templates for that particular deployment:

(5) $$a^{★}\left(x\right)=\arg\max_{a\in M^{12}}\;F\left(x,a\right),$$

where *F* is the loss of the deployment x on case a. This is the inner maximization of the worst-case robustness problem (10) introduced in Section 4. A case generator that searches $M^{12}$ approximates this maximization during training.

### 3.3. Why a Per-Kind Fairness Objective Is Necessary

Single-case optimization uses an aggregate miss-rate penalty in $f_{1}$, expressed as $3\;p_{\mathrm{miss}}$, where $p_{\mathrm{miss}}$ is the overall miss rate. For a case with 12 mixed-kind targets, missing the three T4 targets (one entire kind) and missing three targets of three different kinds both yield $p_{\mathrm{miss}}=0.25$. The aggregate metric is blind to per-kind concentration. The per-kind fairness objective $f_{4}=\max_{k}{\mathrm{MR}}_{k}$, where ${\mathrm{MR}}_{k}$ is the miss rate restricted to kind *k*, separates the two situations. It equals 1 in the first and 1/3 or less in the second. Section 6.4 shows that $f_{4}$ is effective only when the training cases contain the compositions on which a kind can be lost.

### 3.4. Model Scope, Normalized Units and Parameter Set

All lengths and altitudes are expressed in one normalized simulation unit (n.u.), with speeds in n.u./h and time in seconds; κ in (1) is the horizon constant expressed in these units. The model is closed under this normalization. The detection test, confirmation rule and four objectives use only the quantities in Table 2, so every number reported in this paper can be reproduced from that table and the released code without the physical value of the unit. That value is not released, for reasons related to the project under which the study is conducted. The deployment geometry is governed by dimensionless ratios that the table makes explicit. Examples are the reference ranges of the three sensor types relative to the start radius of the targets (100:130:110 against 900) and the horizon reduction of Table 1.

The sensing model is deliberately first-order. It contains the two effects that create the heterogeneous-target problem, the horizon collapse of low flyers and the cross-section dependence of range, and omits clutter, terrain and building masking, multipath, interference, probabilistic detection, false alarms, tracking errors, and environmental attenuation. Five of these omissions are re-introduced as a stochastic sensing model in Section 6.6, where deployments optimized under the first-order model are re-evaluated without re-optimization.

## 4. Problem Formulation

### 4.1. Traffic Model and Objectives

**Traffic model.** A target library M of |M|=35 templates spanning four kinds is defined (Table 2). Kind T1 contributes eight constant-altitude templates and T2 contributes five slower templates with a larger cross section; T3 contributes 12 HL-profile templates that descend to 0.4 n.u. and T4 contributes 10 surface-skimming templates with a much smaller cross section. Each template fixes an approach angle and a launch wave. A case $a\in M^{12}$ is a set of 12 distinct templates.

**Objectives.** The deployment $x\in R^{D}$ encodes the horizontal positions of $N_{s}$ sensors ($D=2N_{s}$). Given a case a, four objectives are computed:

(6) $$\begin{array}{cc} f_{1}\left(x,a\right) & =1-{\bar{R}}_{\det}/R_{\mathrm{start}}+3\;p_{\mathrm{miss}} \end{array}$$

(7) $$\begin{array}{cc} f_{2}\left(x,a\right) & ={\bar{t}}_{\det}/T_{\mathrm{sim}} \end{array}$$

(8) $$\begin{array}{cc} f_{3}\left(x,a\right) & =1-{\bar{d}}_{\mathrm{track}}/T_{\mathrm{sim}} \end{array}$$

(9) $$\begin{array}{cc} f_{4}\left(x,a\right) & =\max_{k}{\mathrm{MR}}_{k}\left(x,a\right) \end{array}$$

where ${\bar{R}}_{\det}$, ${\bar{t}}_{\det}$, ${\bar{d}}_{\mathrm{track}}$ and $p_{\mathrm{miss}}$ are the mean confirmation range, mean confirmation time, mean track duration and overall miss rate, and ${\mathrm{MR}}_{k}$ is the miss rate of kind *k*. All four are minimized; $F=0.4f_{1}+0.4f_{2}+0.2f_{3}$ is the scalar detection score.

**Test metrics.** A deployment is scored on a set of held-out cases by three worst-case metrics: $F_{\max}$, the largest *F* over the cases; $f_{4,\max}$, the largest $f_{4}$; and $n_{\mathrm{bad}}$, the number of cases on which at least one target is missed ($f_{4}>0$). The number of cases on which an entire kind is missed ($f_{4}=1$) is reported as $n_{\mathrm{cat}}$.

### 4.2. Worst-Case Robustness and Game-Theoretic Interpretation

The worst-case robustness objective seeks the $x^{*}$ that performs well on the hardest case:

(10) $$x^{*}=\arg\min_{x\in \Omega }\;\max_{a\in A_{\mathrm{wc}}\left(x\right)}\;F\left(x,a\right)$$

where $A_{\mathrm{wc}}\left(x\right)$ is the set of cases that a case generator may select against deployment x. The fairness objective $f_{4}$ does not enter *F*; it participates in the four-objective selection of the deployment population (via mean_max4, Section 5) and is reported through $f_{4,\max}$.

Equation (10) is the value of a single-stage, two-player, zero-sum game with deployment action $x\in \Omega \subset R^{D}$ and case action $a\in M^{12}$, the degenerate case of a two-player, zero-sum Markov game [44] with a single state and no transition dynamics. Value-based solvers such as minimax-Q learning [44] and policy-space response oracles [45] do not transfer cleanly. The deployment action is continuous (D=4–10), and the case action is a combinatorial selection of 12 templates from 35 with no temporal structure. The saddle point is therefore approximated by population-based co-evolution, in which the hall of fame plays a role analogous to the opponent population of policy-space response oracles.

## 5. The ACE Framework and ACE-A6

### 5.1. The ACE Training Framework

ACE trains a deployment population against a population of traffic cases. Three components define it, independently of the optimizer that updates the deployments:

1. **Kind-diverse case population.** The case population *A* ($n_{a}=5$) is initialized with four composition quotas, {*T*1:3, *T*2:2, *T*3:4, *T*4:3}, {*T*4:6, *T*3:4, *T*1:2}, {*T*1:8, *T*2:2, *T*3:2} and {*T*3:8, *T*4:2, *T*1:2}, so that every kind is over-represented in at least one case. In each generation, the cases are ranked by their fitness against the current non-dominated deployments, $E_{d}\left[F\left(d,a\right)+0.5f_{4}\left(d,a\right)\right]$; the stronger half is kept and the rest is replaced by template-swap mutants of the elite.
2. **Clustered hall of fame.** The strongest new case of each generation enters the hall of fame (HoF), which is clustered by a six-dimensional signature (kind composition and angle statistics) whenever it exceeds $k_{\mathrm{hof}}=6$ members, so that it keeps distinct hard compositions rather than near-duplicates.
3. **Robust aggregation and selection.** A deployment is scored on A∪HoF by mean_max4, the means of $f_{1},f_{2},f_{3}$ and the maximum of $f_{4}$ over the cases, and the final deployment is the candidate that minimizes $F+0.5f_{4}$ when re-evaluated on the accumulated case set.

Any population-based optimizer can update the deployments inside this loop. Section 6.3 places five external optimizers in it.

### 5.2. ACE-A6 and Its Modes

**Figure 1:**
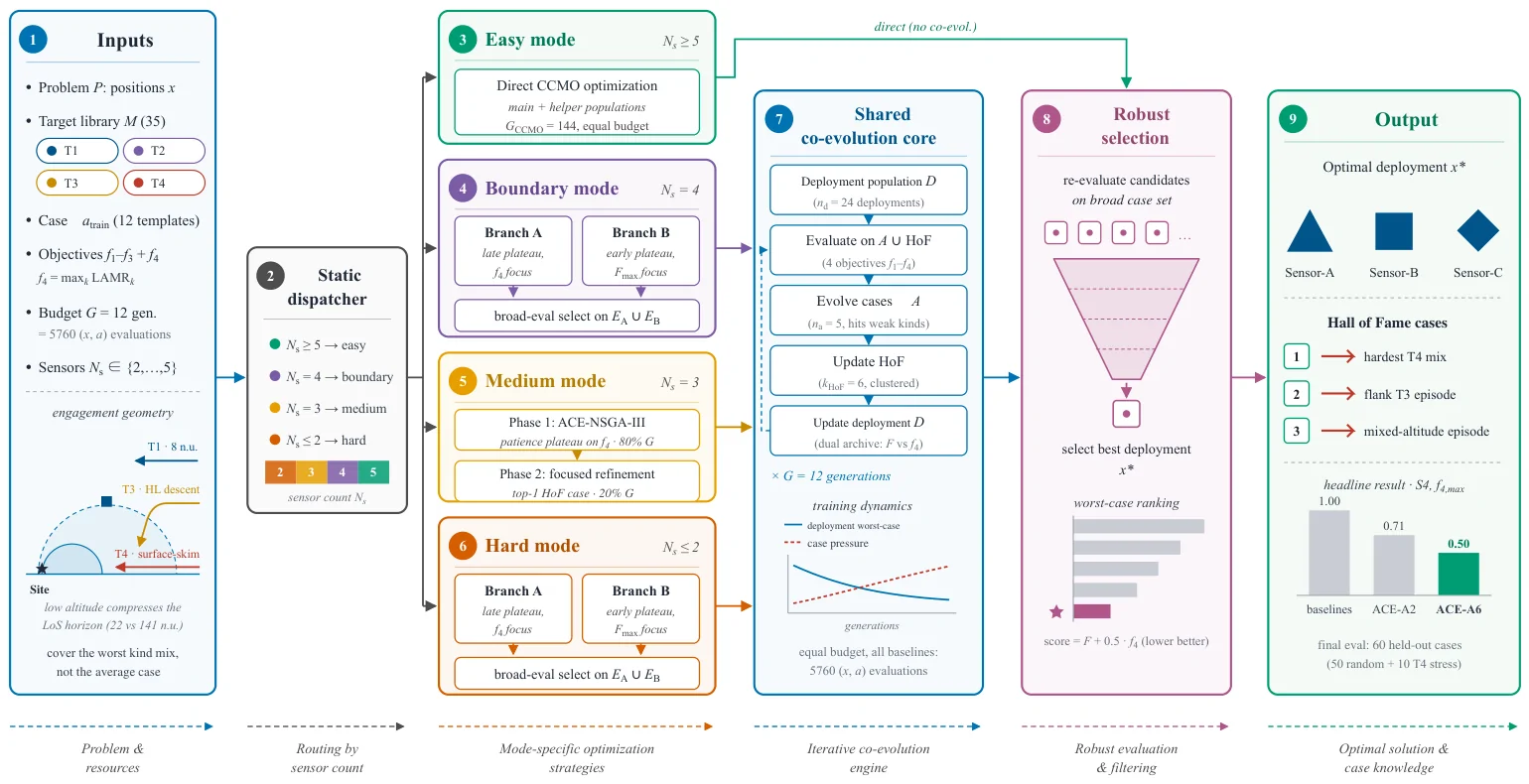
ACE-A6 architecture. The static dispatcher routes the deployment problem P to one of four internal modes based on the sensor count $N_{s}$: Easy mode ($N_{s}\geq 5$, coevolutionary constrained multi-objective optimization (CCMO) on the single training case); Boundary mode ($N_{s}=4$, dual-branch ensemble of a late-plateau and an early-plateau co-evolution branch, selected by broad evaluation on $E_{A}\cup E_{B}$); Medium mode ($N_{s}=3$, a two-generation probe followed by patience-based co-evolution with focused refinement; Section 5.2); or Hard mode ($N_{s}\leq 2$, the same dual-branch ensemble as Boundary mode). All co-evolutionary modes share the adaptive co-evolution (ACE) core, in which the deployment population *D* is evaluated against the case population *A* and the clustered hall of fame, the case population evolves towards compositions the current deployments handle worst, and robust selection re-evaluates the candidates on the broad case set.

Figure 1 shows the architecture of ACE-A6, which dispatches a deployment problem to one of four internal modes based on the sensor count $N_{s}$.

**Co-evolution branch (ACE-A2 core).** The branch primitive runs co-evolution for G=12 generations with a deployment population of $n_{d}=24$. In each generation, every d∈D and every offspring ( simulated binary crossover (SBX) and polynomial mutation, repaired by box clipping and the ordering constraints of Table A1) is evaluated on A∪HoF. A dual archive selects the next population, half by $F+0.5f_{4}$ for convergence and half by nearest-neighbor distance in objective space for diversity, and the case population and HoF are then updated as above. Phase 1 ends after 80% of the generations or earlier by a plateau rule. Phase 2 refines the population on the hardest HoF case, and a cross-stage selection re-evaluates the Phase 2 candidates on the Phase 1 case set.

**Boundary (**$N_{s}=4$**) and Hard (**$N_{s}\leq 2$**) modes** run two such branches with different plateau rules. Branch A ends Phase 1 after three generations without a new maximum of case fitness (late plateau, favoring $f_{4}$) and branch B ends it when the relative growth of the case fitness over three generations falls below 1% (early plateau, favoring $F_{\max}$). Each branch has the full generation budget, so the mode can spend up to twice the evaluations of a single branch ( Section 6.1). The final deployment is the branch winner under $F+0.5f_{4}$ re-evaluated on the union $E_{A}\cup E_{B}$ of the Phase 1 case sets; on S4, branch B wins on 18 of the 30 seeds and branch A on 12.

**Medium mode (**$N_{s}=3$**)** first runs two co-evolution generations as a probe and reads the largest case fitness found. Below 0.85, the remaining budget goes to CCMO on the training case. Between 0.85 and 1.05, Phase 1 continues with the patience rule (K=3 generations without a new high of case fitness, tolerance 0.005) and is followed by focused refinement. Above 1.05, a single ACE-A2 branch runs. On S3, these three outcomes occur on 2, 22 and 6 of the 30 seeds.

**Easy mode (**$N_{s}\geq 5$**)** bypasses co-evolution and runs CCMO [30] on the single training case with the standard configuration of the baseline (40 individuals, 144 generations). CCMO evolves two populations, one on the original problem and one on a helper problem whose miss-rate penalty is relaxed, and exchanges offspring between them.

**Design rationale.** The modes follow from the coverage capacity of the network. With five sensors, the combined coverage of the scenario geometry exceeds what the library can exploit, a single training case already exposes the relevant blind spots and co-evolution adds evaluations without information. With four or fewer sensors, the deployment must trade coverage between angular sectors, small position changes decide which kind is missed and only training on several hard compositions exposes those blind spots. The two plateau rules over-fit on largely disjoint seed subsets, which is why the ensemble selects between them on the union of their case sets. Section 6.5 tests whether this sensor-count rule transfers to scenarios outside its design set.

**Figure 2:**
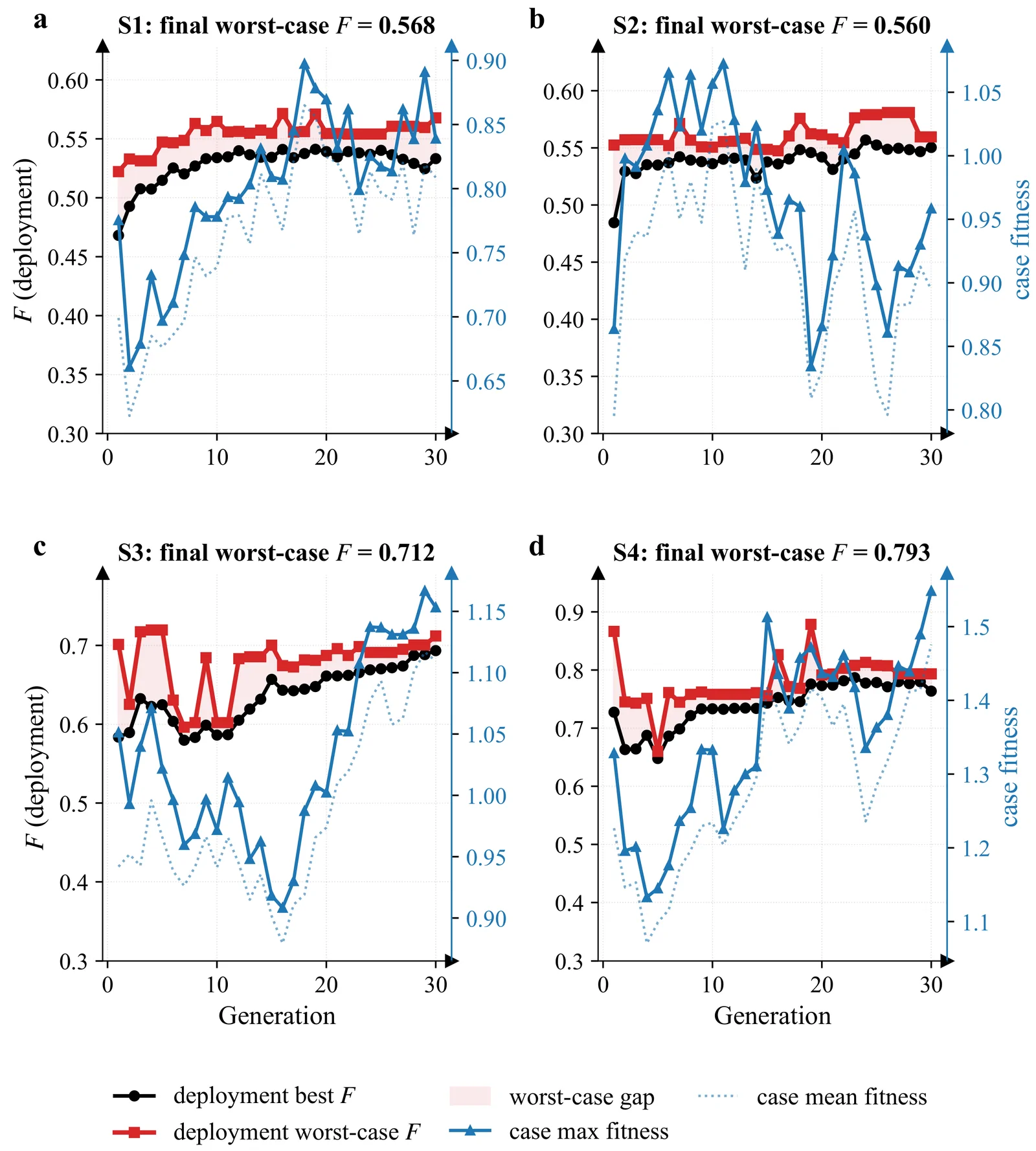
Co-evolutionary dynamics on the four scenarios: (**a**) S1; (**b**) S2; (**c**) S3; (**d**) S4. Each panel shows the best deployment score *F* (black), the worst-case *F* of the same deployment over the current case set (red) and the case fitness on the right axis (blue). The panel title reports the final worst-case *F*; the hall of fame (HoF) holds six cases at the end of every run. The final worst-case level rises with scenario difficulty (S1, S2 < S3 < S4).

**Convergence.** ACE belongs to the competitive co-evolutionary algorithms studied formally in [46]. Convergence to a saddle point of (10) is not guaranteed in general; cycling [47] and disengagement [48] are well documented. Three standard remedies [49] mitigate them. HoF retention prevents the deployments from forgetting hard past cases, HoF clustering prevents the case population from collapsing onto similar cases and the cross-stage broad evaluation keeps the reported deployment robust on the whole Phase 1 case set. Figure 2 shows the case fitness rising across generations and a persistent gap between the training score and the worst case.

**Pseudocode.** The dispatcher (Algorithm 1) routes the problem to a mode; Algorithm 2 details the co-evolution branch, which the Boundary and Hard modes instantiate twice.
**Algorithm 1** ACE-A6 top-level dispatcher**Require:** Deployment problem P with $N_{s}$ sensors; target library M; training case $a_{\mathrm{train}}$; budget *G*
1:**if** $N_{s}\geq 5$ **then**2:   $x^{*}\leftarrow \text{run\_CCMO}\left(P{|}_{a_{\mathrm{train}}}\right)$ {Easy mode}3:**else if** $N_{s}=4$ **then**4:   $x^{*}\leftarrow \text{run\_ACE\_dual\_branch}\left(P,M,G\right)$ {Boundary mode}5:**else if** $N_{s}=3$ **then**6:   c← largest case fitness of a 2-generation probe7:   $x^{*}\leftarrow$ CCMO if c<0.85; patience co-evolution if 0.85≤c<1.05; one ACE-A2 branch otherwise {Medium}8:**else**9:   $x^{*}\leftarrow \text{run\_ACE\_dual\_branch}\left(P,M,G\right)$ {Hard mode}10:**end if**11:**return** $x^{*}$


**Algorithm 2** Co-evolution branch (ACE-A2 core)**Require:** Problem P, library M; budget *G*; population sizes $n_{d},n_{a}$; HoF size $k_{\mathrm{hof}}$; plateau rule
1:Initialize deployment population *D* ($n_{d}$ random feasible deployments)2:Initialize case population *A* ($n_{a}$ cases from the four composition quotas)3:HoF←∅; $G_{1}\leftarrow \left\lceil 0.80G\right\rceil$4:**for** generation g=1 to $G_{1}$ **do**5:   E←A∪HoF6:   Score *D* and its offspring on E by mean_max47:   $D\leftarrow \mathrm{DualArchiveSelect}(D\cup D^{\prime },n_{d})$8:   Evolve *A* against the non-dominated deployments; add the strongest new case to HoF; cluster if $|\mathrm{HoF}|>k_{\mathrm{hof}}$9:   **if** plateau rule fires **then**10:     **break**11:   **end if**12:**end for**13:${\mathrm{HoF}}_{\mathrm{top}}\leftarrow$ hardest case in HoF14:**for** the remaining generations (Phase 2) **do**15:   Evaluate *D* against ${\mathrm{HoF}}_{\mathrm{top}}$; $D\leftarrow \mathrm{DualArchiveSelect}(D\cup \mathrm{offspring}\left(D\right),n_{d})$16:**end for**17:Re-evaluate the Phase 2 candidates on E and return the minimizer of $F+0.5f_{4}$


## 6. Experimental Results

### 6.1. Experimental Setup

**Figure 3:**
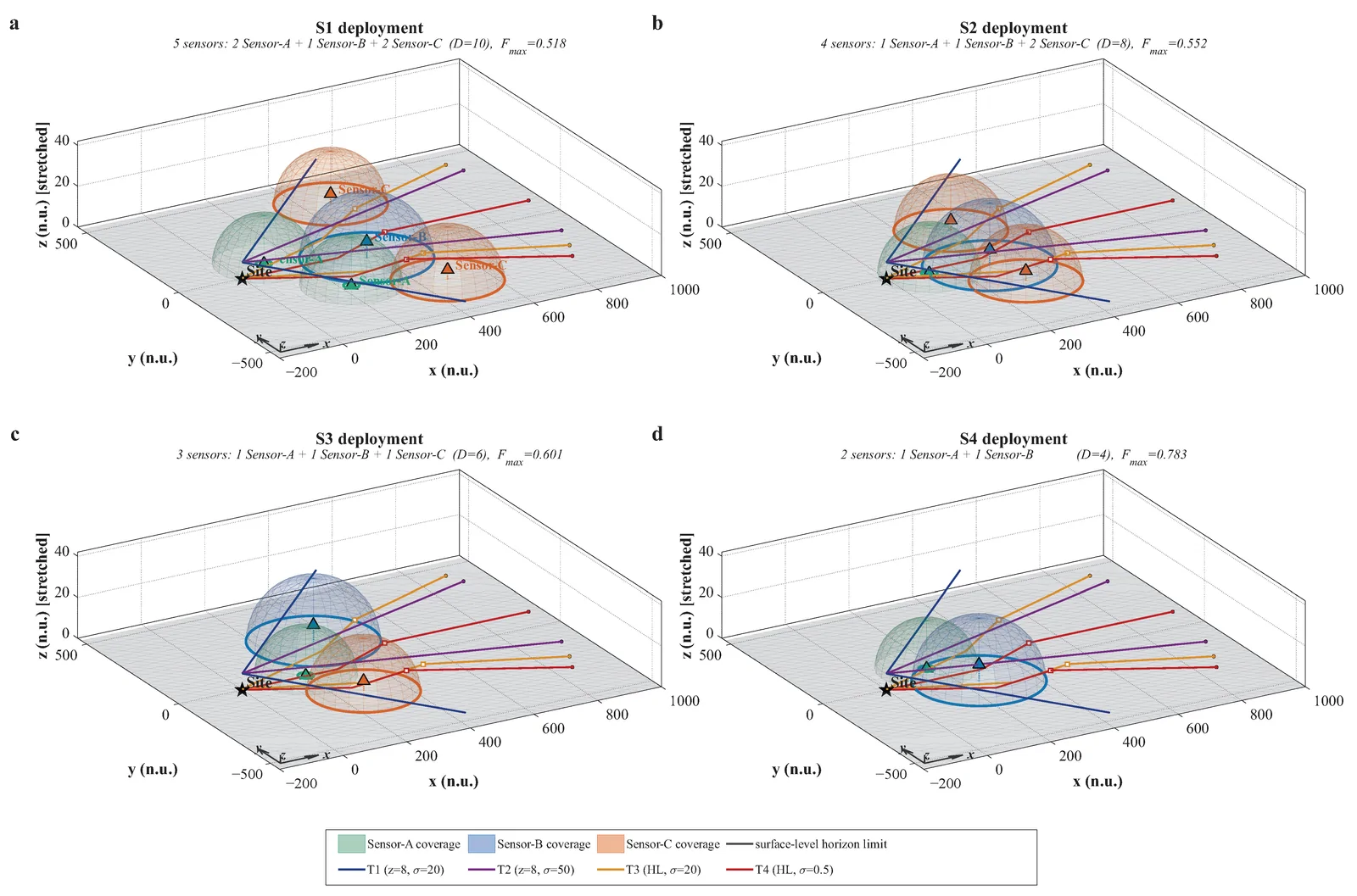
The four deployment scenarios, with the best-of-30-seed ACE-A6 deployment in each; the panel title gives its worst-case score $F_{\max}$ on the 60 held-out cases. The overlaid target library shows T1 (deep blue, $h_{t}=8$ n.u., σ=20), T2 (purple, $h_{t}=8$ n.u., σ=50), T3 (gold, high–low transition (HL), $h_{t}$: 8→0.4 n.u., σ=20) and T4 (red, HL, $h_{t}$: 8→0.05 n.u., σ=0.5); the site (black star) is at the origin. The coverage of each sensor is drawn as a dome at $R_{\mathrm{eff}}\left(h_{s},h_{t}=8\;n.u.,\sigma =20\right)$, and the surface ring marks its horizon-limited footprint at $h_{t}=0$ (∼22 n.u. for Sensor-A). The vertical axis is stretched 6×. (**a**) **S1**, $F_{\max}=0.518$, 2 Sensor-A + 1 Sensor-B + 2 Sensor-C, D=10. (**b**) **S2**, $F_{\max}=0.552$, 1 Sensor-A + 1 Sensor-B + 2 Sensor-C, D=8. (**c**) **S3**, $F_{\max}=0.601$, 1 Sensor-A + 1 Sensor-B + 1 Sensor-C, D=6; Sensor-B is pushed to y=244 n.u. and Sensor-C to −y. (**d**) **S4**, $F_{\max}=0.783$, 1 Sensor-A + 1 Sensor-B, D=4.

**Scenarios.** Four deployment scenarios with $N_{s}\in \left\{5,4,3,2\right\}$ sensors are evaluated (S1–S4, Figure 3); their placement boxes and ordering constraints are listed in Table A1. Each method is run with 30 random seeds (42–71).

**Methods.** The external methods are Random Search (RS), NSGA-II, PW-NSGA-II (an enhanced NSGA-II with priority-weighted dominance, Latin hypercube initialization, adaptive Gaussian mutation, elite local search and diversity restarts) and NSGA-III with four objectives and the state-of-the-art algorithms CCMO [30], C-TAEA [29], MaOEA-PDE [24] and EnMOGKSO [6]. All are Python re-implementations that follow the published designs. The helper problem of CCMO relaxes the miss-rate penalty of $f_{1}$ by subtracting 0.5 (floored at 0); C-TAEA, CCMO and NSGA-II optimize $\left(f_{1},f_{2},f_{3}\right)$ and NSGA-III, MaOEA-PDE and EnMOGKSO optimize all four objectives. All external methods are trained on the single training case, as in their original formulation. The ACE family comprises ACE-A6 and the ablation variants of Section 6.7.

**Evaluation budget.** The budget is counted as the number of (deployment, case) evaluations during training, measured by instrumenting the evaluation routine. Table 3 reports the medians over the 30 seeds. CCMO evaluates two populations per generation and ACE-A6 spends an amount that depends on its mode and plateau rules, so the budgets are not equal. The comparison that controls for budget is made in Section 6.3, following the guidelines of [50].

**Hyperparameters.** ACE uses $n_{d}=24$, $n_{a}=5$, $k_{\mathrm{hof}}=6$, G=12, focused refinement on the top-1 HoF case, patience 3 with tolerance 0.005, SBX ($\eta_{c}=20$) and polynomial mutation ($\eta_{m}=20$, probability 1/D). The external baselines use 40 individuals and 144 generations; NSGA-III uses Das–Dennis reference points with p=4 divisions (35 points for 4 objectives).

**Training and test cases.** Training uses a single case with quota {*T*1:3, *T*2:2, *T*3:4, *T*4:3} (RNG seed 123), identical for all methods. The primary test set has 60 held-out cases (random number generator (RNG) seed 999), namely, 50 unconstrained random cases and 10 T4-heavy stress cases with quota {*T*4:5, *T*3:4, *T*1:3}. A second, independent test set generated in the same way with RNG seed 2026 is new in this revision. It guards against conclusions that depend on the particular test cases used during method development.

**Statistical protocol.** Comparisons follow the nonparametric protocol recommended for stochastic optimizers [51]. They use paired Wilcoxon signed-rank tests [52] (two-sided, seeds paired) with Cliff’s delta δ [53] as effect size (negligible <0.147, small <0.33, medium <0.474, large otherwise [54]). Table 4 and Table 5 use the Bonferroni correction over the K=180 comparisons of the original study. The analyses added in this revision use the Holm correction within each scenario over all comparisons of the respective table. A method is reported as better only if the corrected p<0.05 and the effect has the stated direction.

**Platform and reproducibility.** The original experiments run single-threaded in Python 3.11 with NumPy on an Intel i9-13900K workstation, and one (deployment, case) evaluation takes about 0.5 ms. For this revision, 192 runs (all 16 methods, 4 scenarios, seeds 42–44) are repeated on a second Windows workstation (Intel Core Ultra 9 285K, Python 3.13.5, NumPy 2.1.3) and reproduce the archived test metrics exactly. All 1920 runs are also regenerated on a Linux server (Python 3.13.16, NumPy 2.1.3). Of these, 1891 reproduce exactly, and the remaining 29 diverge through floating-point differences between the platforms. The experiments added in Section 6.3, Section 6.4, Section 6.5 and Section 6.6 run on the Linux server.

### 6.2. Comparison with Published Baselines

Table 4 summarizes the median test set metrics of the external methods and ACE-A6 over 30 seeds; the full table with all ACE variants is Table A2.

**Figure 4:**
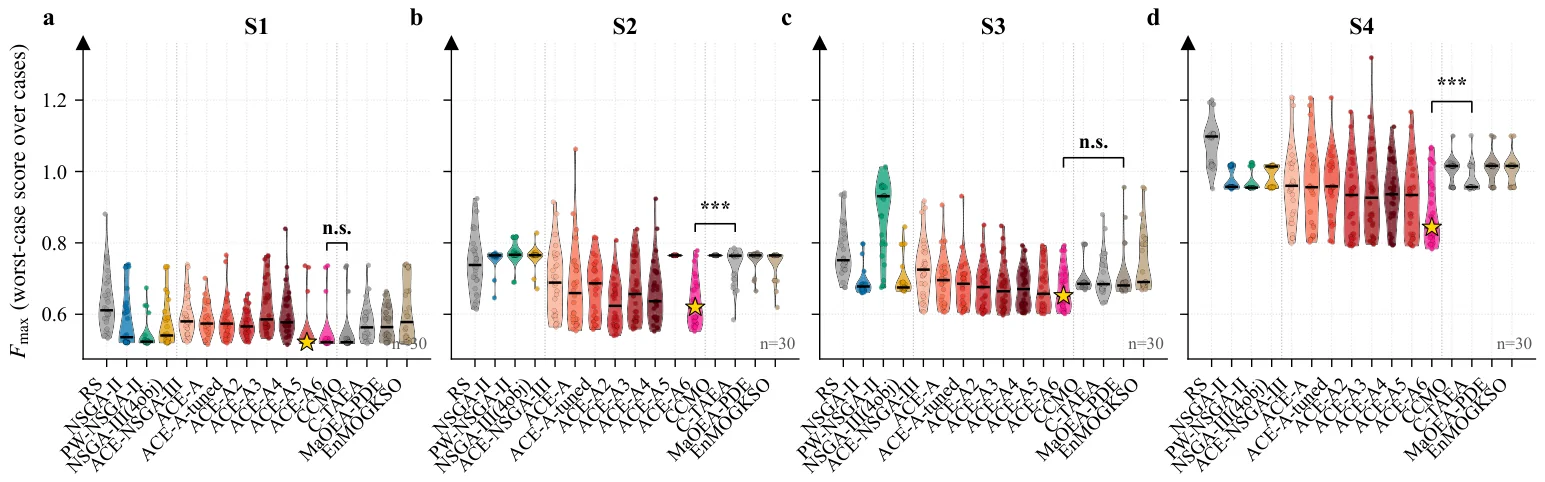
Per-seed worst-case score $F_{\max}$ of the 16 methods on the four scenarios (30 seeds): (**a**) S1; (**b**) S2; (**c**) S3; (**d**) S4. ACE-A6 attains the lowest median on S1 (tied with CCMO, which it runs in Easy mode), S2, S3 and S4. Black bar = median; gold ⋆ = best median per scenario; brackets report the paired Wilcoxon test of ACE-A6 against the best state-of-the-art baseline (***, $p_{\mathrm{Bonf}}<10^{-3}$; n.s., not significant, $p_{\mathrm{Bonf}}\geq 0.05$; $p_{\mathrm{Bonf}}$, Bonferroni-corrected *p*).

**S1 (five sensors).** ACE-A6 runs CCMO in Easy mode and therefore reproduces CCMO exactly ($F_{\max}=0.522$, Figure 4).

**Figure 5:**
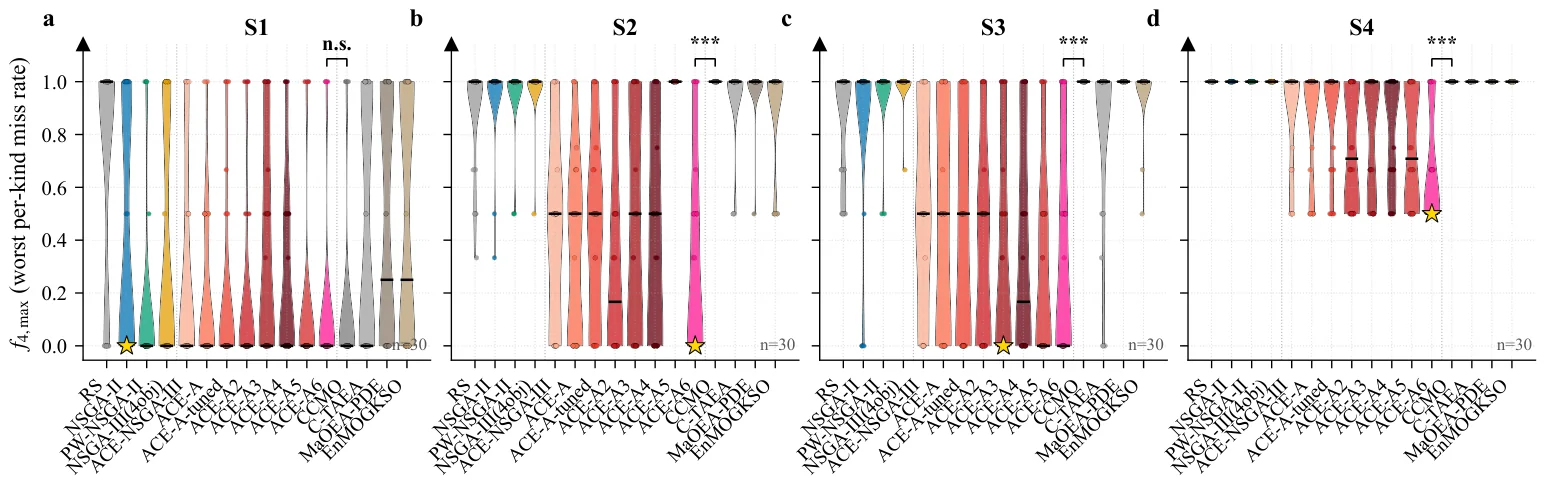
Per-seed worst per-kind miss rate $f_{4,\max}$ of the 16 methods on the four scenarios (30 seeds): (**a**) S1; (**b**) S2; (**c**) S3; (**d**) S4. The eight external methods cluster at $f_{4,\max}=1$ on S2–S4. Black bar = median; gold ⋆ = best median per scenario; brackets report the paired Wilcoxon test of ACE-A6 against the best state-of-the-art baseline (***, Bonferroni-corrected $p_{\mathrm{Bonf}}<10^{-3}$; n.s., not significant, $p_{\mathrm{Bonf}}\geq 0.05$).

**S2 (four sensors).** Every external method reaches $f_{4,\max}=1$, so on some test cases, a whole kind is missed. ACE-A6 attains median $F_{\max}=0.620$ and $f_{4,\max}=0$ (δ≥+0.75, $p_{\mathrm{Bonf}}\leq 2.9\times 10^{-3}$ against all eight external methods; Table 5, Figure 5).

**S3 (three sensors).** ACE-A6 attains the lowest median $F_{\max}=0.652$ and $f_{4,\max}=0$, against $f_{4,\max}=1$ for every external method (Table 5).

**S4 (two sensors).** ACE-A6 attains median $F_{\max}=0.844$ and $f_{4,\max}=0.500$; all external methods reach $f_{4,\max}=1$.

**Figure 6:**
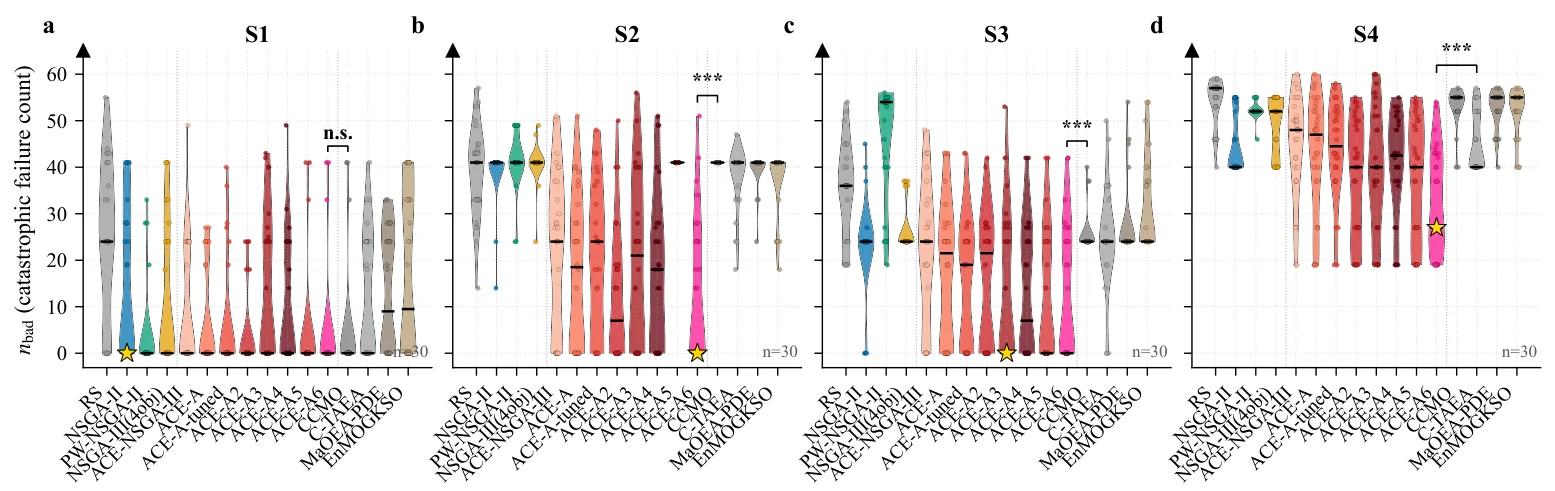
Per-seed $n_{\mathrm{bad}}$, the number of the 60 test cases on which at least one target is missed ($f_{4}>0$): (**a**) S1; (**b**) S2; (**c**) S3; (**d**) S4. ACE-A6 attains the lowest median in all four scenarios. Black bar = median; gold ⋆ = best median per scenario; brackets report the paired Wilcoxon test of ACE-A6 against the best state-of-the-art baseline (***, Bonferroni-corrected $p_{\mathrm{Bonf}}<10^{-3}$; n.s., not significant, $p_{\mathrm{Bonf}}\geq 0.05$).

**Figure A1:**
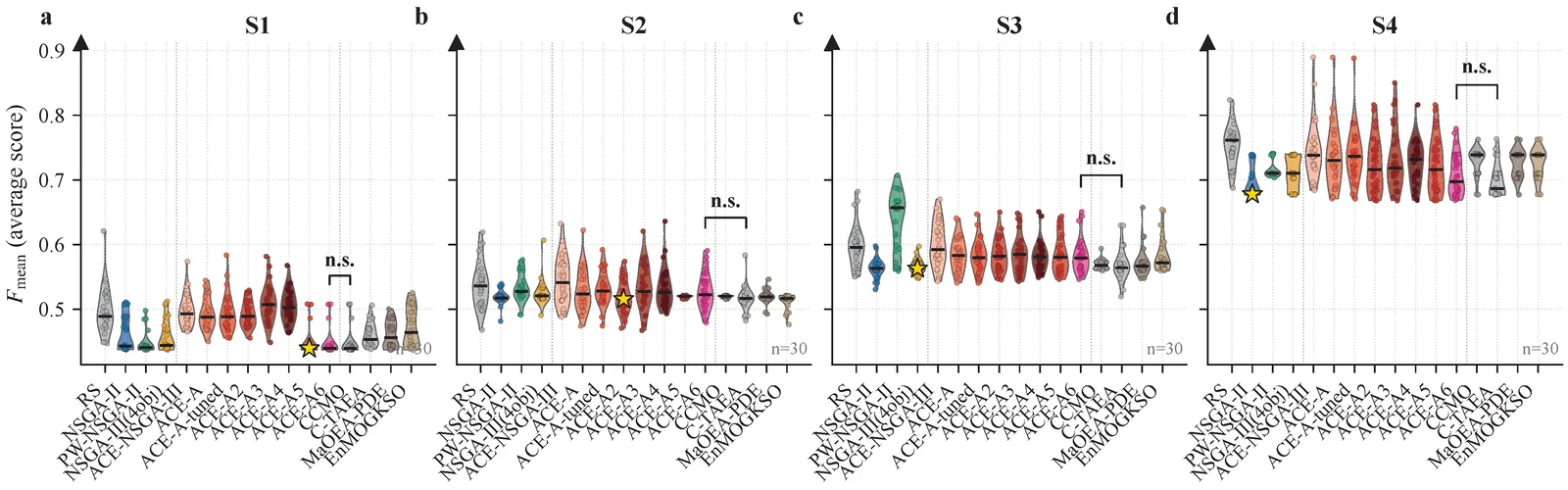
Per-seed average score $F_{\mathrm{mean}}$ over the 60 test cases (lower is better): (**a**) S1; (**b**) S2; (**c**) S3; (**d**) S4. Black bar = median; gold ⋆ = best median per scenario; brackets report the paired Wilcoxon test of ACE-A6 against the best state-of-the-art baseline (n.s., not significant, Bonferroni-corrected $p_{\mathrm{Bonf}}\geq 0.05$).

**Miss counts.** On the primary test set, the median $n_{\mathrm{bad}}$ of ACE-A6 is 0, 0, 0 and 27 on S1–S4 (Figure 6), against 41 on S2 and 24 on S3 for every state-of-the-art baseline and 40 (C-TAEA, NSGA-II) to 55 (CCMO, MaOEA-PDE, EnMOGKSO) on S4. The median number of cases on which a whole kind is missed, $n_{\mathrm{cat}}$, is 0 for ACE-A6 on every scenario and 1 to 3 for the state-of-the-art baselines on S2–S4. The median $F_{\mathrm{mean}}$ of ACE-A6 equals that of CCMO on S1 and lies within 3% of the lowest median on S3 (Figure A1), so the average score is not traded for these gains.

**Independent test set.** On the second test set (seed 2026), the conclusions are unchanged. ACE-A6 attains median $F_{\max}$ of 0.513, 0.605, 0.646 and 0.808 and median $f_{4,\max}$ of 0, 0, 0 and 0.5 on S1–S4. Against each of the four state-of-the-art baselines on S2–S4, it is better on $F_{\max}$ (δ 0.56–0.92) and $f_{4,\max}$ (δ 0.44–0.80), all with Holm-corrected p≤0.037.

### 6.3. Same-Framework Comparison at Equal Budget

The comparison above confounds two differences between ACE-A6 and the baselines: the optimizer and the training framework (one fixed case versus a co-evolved population of kind-diverse cases). To separate them, five external optimizers (CCMO, C-TAEA, MaOEA-PDE, EnMOGKSO and NSGA-II) are placed inside the ACE framework with their own operators unchanged. In the co-evolved arm, the optimizer replaces the ACE-A2 branch. It runs for a few generations on the mean_max4 aggregate over A∪HoF, warm-started from its previous population, and the case population and HoF are then updated against its non-dominated set for up to 12 rounds. In the static arm, the case set is fixed at 11 cases, drawn once with the same composition quotas, without a case generator. Both arms use the ACE robust final selection and stop as soon as the next round no longer fits within a measured budget equal to that of ACE-A6. That budget comes from a three-seed pilot (S2 9486, S3 2730, S4 6197 evaluations), and the comparison is repeated below with the 30-seed medians of Table 3. The static and co-evolved arms also run at 11,600 evaluations, the largest native budget.

**Figure 7:**
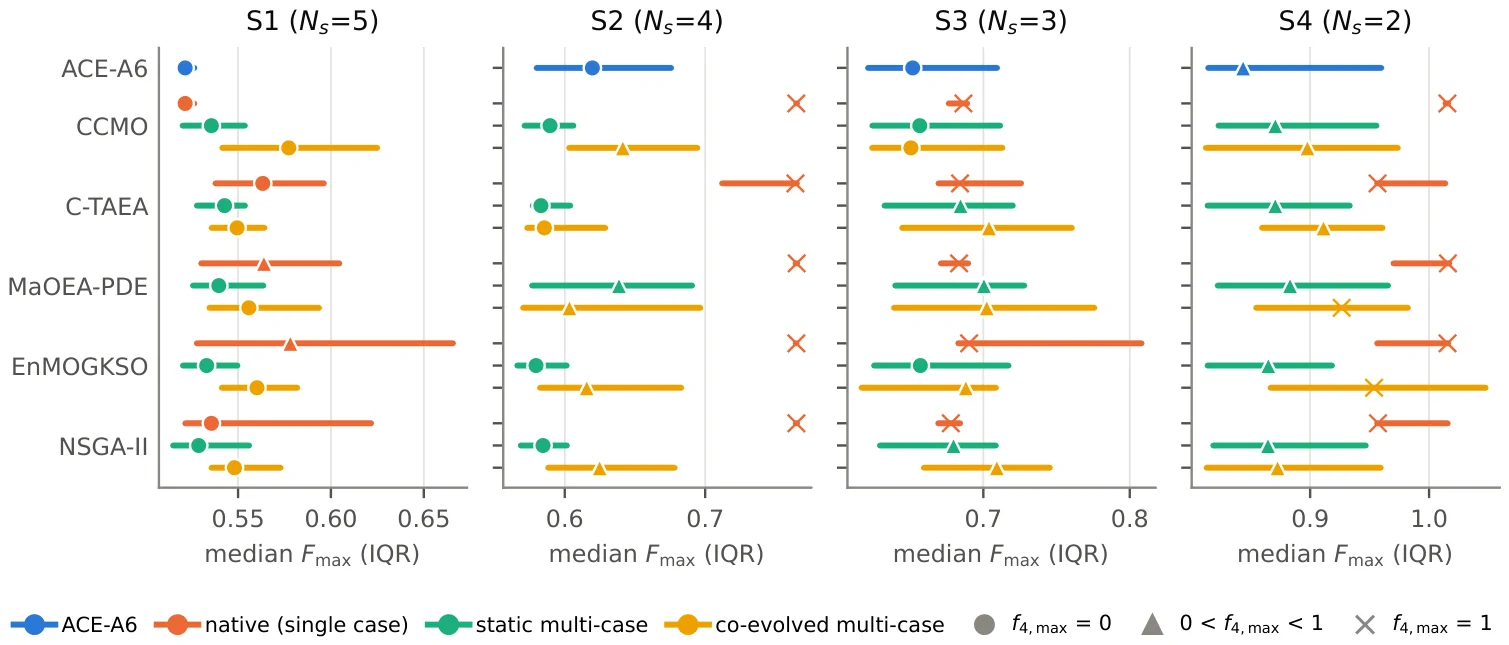
Same-framework comparison at the ACE-A6 budget (primary test set, 30 seeds). Each of five optimizers appears under native single-case training (orange), static multi-case training (aqua) and co-evolved multi-case training inside ACE (yellow), with ACE-A6 in blue. Dots mark the median $F_{\max}$, bars the interquartile range, and the marker shape the median $f_{4,\max}$ (circle 0, triangle between 0 and 1, cross 1).

Table 6 and Figure 7 give three results. First, the training framework breaks the ceiling. Inside ACE, every optimizer leaves $f_{4,\max}=1$ behind on S2 and S3, and at 11,600 evaluations, all five reach a median of 0 there. Second, at the ACE-A6 budget, no comparison between ACE-A6 and an optimizer inside the framework is significant on S2–S4. ACE-A6 has the lowest median $F_{\max}$ on S4 and ties the best arm on S3, while on S2, the static arms of four optimizers and the co-evolved C-TAEA have lower medians. Third, with 1.5 to 4.3 times the ACE-A6 budget, the framework arms improve further, and one arm becomes significantly better than ACE-A6 on S2 and one on S3. Repeating the S2 and S4 comparison at the 30-seed median budgets of Table 3 (7866 and 7225 evaluations) gives the same picture on both test sets. No difference from ACE-A6 is significant; ACE-A6 keeps the lowest median $F_{\max}$ on S4 (0.844 against 0.859–0.927), and on S2, six static or co-evolved arms reach median $F_{\max}$ between 0.581 and 0.594 with $f_{4,\max}=0$. Because an arm stops as soon as the next round no longer fits within its budget, the arms use 5720 to 7800 evaluations in this repetition, 1% to 27% fewer than ACE-A6. On the independent test set, the same pattern holds; the only significant difference at the ACE-A6 budget is a lower $F_{\max}$ of static EnMOGKSO on S2 (δ=−0.44, Holm p=0.031). ACE is therefore best read as a training framework that lifts any of these optimizers, and ACE-A6 as an instance that is competitive inside it, with the clearest margin on the two-sensor scenario.

### 6.4. Contribution of the Fairness Objective and Training Cases

A 2×3 factorial isolates the two ingredients. One optimizer (NSGA-III, 36 individuals) and one budget (5800 evaluations) are used throughout. The objective set is the first factor. With $f_{4}$, the arm optimizes four objectives and uses $F+0.5f_{4}$ as case fitness and final selection score, and without $f_{4}$, it optimizes three objectives and uses *F* alone. The training cases are the second factor, namely, the single training case, the static set of 11 cases, or the co-evolved case set.

Table 7 replaces the single-scenario 2×2 table of the original submission. The training cases dominate. With one training case, $f_{4,\max}$ stays at 1 whether or not $f_{4}$ is optimized, and moving to several kind-diverse cases lowers it in every scenario. Replacing the single case by the static or co-evolved case set lowers $f_{4,\max}$ significantly in all 16 combinations of scenario, objective set and case set (δ 0.34–0.91, Holm p≤0.024 over the 32 comparisons of this kind). Given several training cases, adding $f_{4}$ lowers the median $f_{4,\max}$ in five of the six multi-case comparisons on S2–S4 (for example, from 0.50 to 0 on S2 and from 1.00 to 0.67 with co-evolution on S4). None of these differences survive the Holm correction over the 24 comparisons, and on S3 with co-evolution, the arm without $f_{4}$ is better. The fairness objective therefore acts as a selection criterion that converts the information carried by kind-diverse cases into lower per-kind miss rates; it cannot create that information when the deployment sees a single case.

### 6.5. Generalization of the Dispatch Rule

The sensor-count rule is designed on S1–S4. Ten held-out scenarios (G1–G10, Table 8) vary the sensor mixture at a fixed count, the count itself ($N_{s}=6$ and $N_{s}=1$), the placement region and the target library. On each, the three distinct mode algorithms of ACE-A6 (Easy, Medium and the dual-branch ensemble shared by Boundary and Hard) run with 30 seeds, and the mode chosen by the rule is compared with the best mode (the oracle) by the median of $F_{\max}+0.5f_{4,\max}$.

The rule picks the oracle mode on 4 of the 10 scenarios and is never significantly worse than the oracle. Its regret is small except on G3, a three-sensor network without the high-altitude Sensor-B, where the dual-branch ensemble avoids the per-kind failures that the Medium mode leaves ($f_{4,\max}$ 0 against 0.5; regret 0.32, $p_{\mathrm{Holm}}=0.062$). The rule thus transfers in the weak sense that its choices cost little, but the sensor count is only a proxy. The right mode depends on the coverage capacity of the network relative to the library, and that capacity depends on the sensor mixture as well as the count. On the scenarios where every mode leaves $f_{4,\max}=1$ (G4, G6, G8, G10), no dispatch rule can help because the network cannot cover the library. A dispatch-free variant that trains on a fixed set of kind-diverse anchor cases together with the co-evolved cases is examined in Appendix C.

### 6.6. Robustness to Non-Ideal Sensing and Library Shift

**Stochastic sensing.** The deployments of all methods are re-evaluated, without re-optimization, under a stochastic sensing model with five switchable effects. Probabilistic detection draws a Bernoulli variable per sensor, target and step with $P_{d}=1/\left(1+\exp\left(\left(r/R_{\mathrm{eff}}-1\right)/0.08\right)\right)$, so that $P_{d}=0.5$ at the nominal radius. Terrain, clutter and building masking blocks every 10° azimuth sector of every sensor with probability 0.15 for targets below 1 n.u. Environmental attenuation scales the cross-section-limited range by a factor drawn from U(0.8,1) per realization. Sensor position error adds $N(0,5^{2})$ n.u. per coordinate. Track confirmation uses a 3-of-5 rule, with spurious detections at a rate of 0.01 per sensor, target and step. Each effect is applied alone and all five together, at the stated level and at a harsh level (transition width 0.15, masking probability 0.30, attenuation U(0.6,1), position error 15 n.u., 4-of-6 confirmation, false-alarm rate 0.02), with 20 realizations per test case. The metrics are formed from the expected *F* and $f_{4}$ per case.

**Library shift.** Eight shifted libraries generate new test cases. They scale the cross sections by +3 dB and −3 dB, scale the speeds by ±20%, redraw the launch times uniformly in [0,300] s, let the HL kinds descend to half the altitude and 100 n.u. earlier, widen the approach sector from $\pm 55^{\circ }$ to about $\pm 80^{\circ }$, and add an unseen kind T5 (cross-section 0.1, constant altitude 0.15 n.u., speed 300 n.u./h) to the library and test cases.

**Figure 8:**
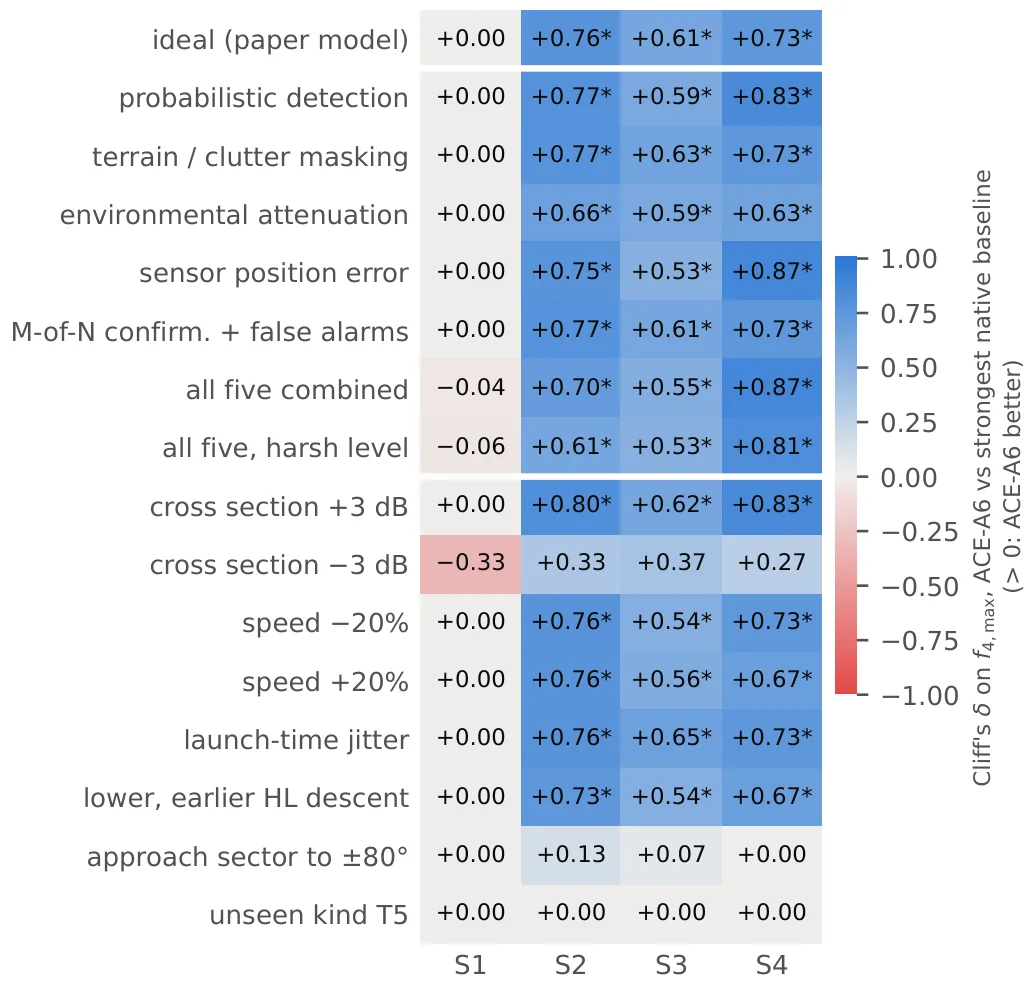
Robustness of the advantage of ACE-A6 (30 seeds). Each cell gives Cliff’s δ on $f_{4,\max}$ of ACE-A6 against the strongest of the four state-of-the-art baselines (the minimum over CCMO, C-TAEA, MaOEA-PDE and EnMOGKSO); * marks Holm-corrected p<0.05 against all four. The rows cover the ideal model, the five non-ideal sensing effects alone and combined (at the stated and at a harsh level), and eight library shifts. S1 is 0 by construction where ACE-A6 runs CCMO.

Figure 8 shows that every non-ideal sensing effect, alone or combined and at either level, leaves the advantage of ACE-A6 on S2–S4 large and significant (δ 0.53–0.87). All methods degrade, with the most degradation occurring under environmental attenuation. For each single effect, the ranking of the methods by median $F_{\max}$ stays close to that under the ideal model (Kendall τ from 0.31 to 0.93, above 0.58 except for attenuation on S4). With all effects combined at the harsh level, it changes substantially (τ from −0.09 to 0.58). Moderate library shifts (cross-section +3 dB, speed ±20%, launch jitter, a lower and earlier HL descent) leave the advantage intact as well. Three severe shifts remove it. With cross-sections reduced by 3 dB, the advantage is no longer significant and the median $f_{4,\max}$ of ACE-A6 rises to 0.75–1. With an approach sector widened to $\pm 80^{\circ }$ or with the unseen kind T5, every method, ACE-A6 included, misses an entire kind on some test cases. These shifts leave the training library, and they mark the condition under which the conclusions hold, namely, that the library covers the approach sectors and target kinds expected in operation. The ACE framework places no restriction on the library content, so such kinds can be added to it.

### 6.7. Ablation and Deployment Geometry

**Figure A2:**
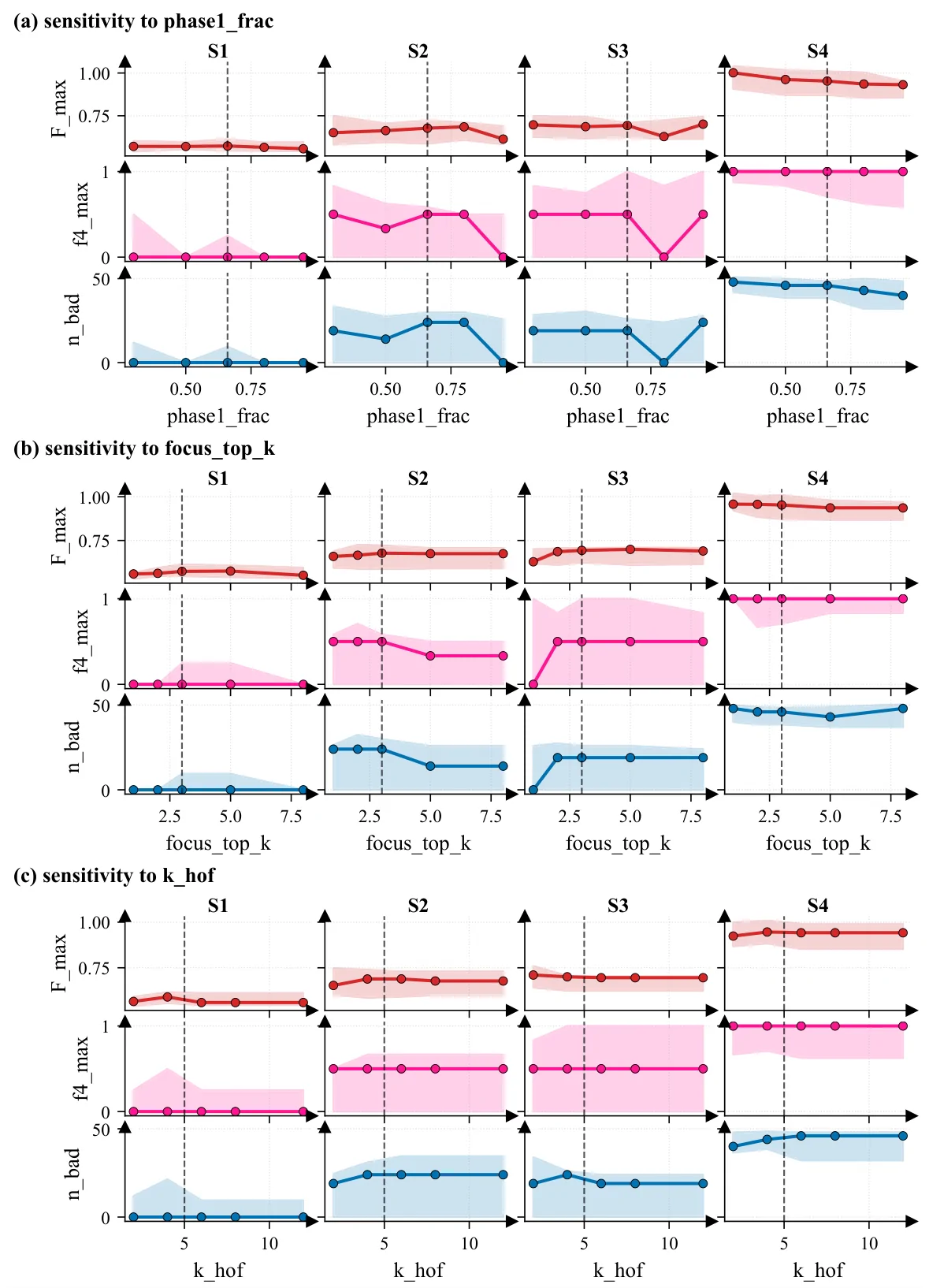
One-at-a-time hyperparameter sensitivity of ACE-A4 (15 seeds per point; red: $F_{\max}$ median; orange: $f_{4,\max}$; blue: $n_{\mathrm{bad}}$; interquartile range (IQR) shaded). (**a**) Phase 1 budget fraction (dashed line: default 0.80). (**b**) Number of HoF cases used in Phase 2 (default 1). (**c**) HoF size; performance plateaus at $k_{\mathrm{hof}}\geq 6$.

**ACE family.** Table 9 traces the ACE variants on S3. Co-evolution and the four-objective selection (ACE-NSGA-III) halve $f_{4,\max}$ from the single-case level of 1.0 to 0.5; the two-phase schedule, tuned hyperparameters, clustered HoF, and dual archive lower $F_{\max}$; and the plateau rules bring $f_{4,\max}$ to 0. The hyperparameter sensitivity of the plateau-based variant is reported in Appendix B, Figure A2.

**Figure 9:**
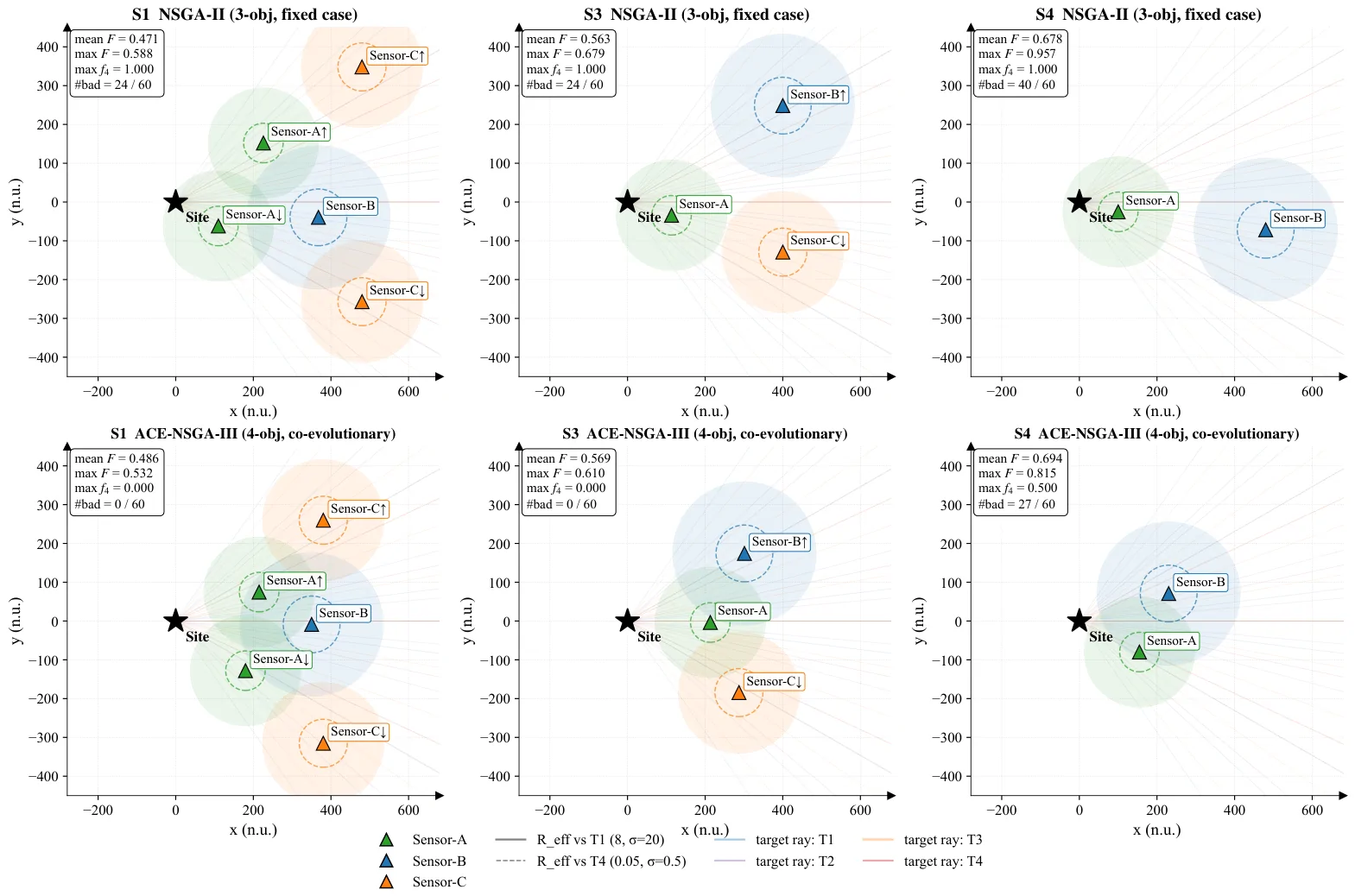
Deployments under single-case and worst-case training. The top row shows NSGA-II on the single training case and the bottom row ACE-NSGA-III under co-evolutionary training for S1, S3 and S4 from left to right. Detection circles show $R_{\mathrm{eff}}$ against a T1 target (solid, $h_{t}=8$ n.u., σ=20) and a T4 target (dashed, $h_{t}=0.05$ n.u., σ=0.5); rays from the origin show the library angles per kind. An arrow after a sensor name marks the sensor whose placement box lies above (↑) or below (↓) the centerline; arrowheads at the axis ends mark the positive axis directions; #bad is $n_{\mathrm{bad}}$. On S3, co-evolutionary training separates Sensor-B and Sensor-C to cover the $\pm 50^{\circ }$ flanks at the cost of about 100 n.u. of forward depth; on S4, it breaks the centerline symmetry of NSGA-II, placing Sensor-B 71 n.u. above and Sensor-A 80 n.u. below the centerline.

**Figure A3:**
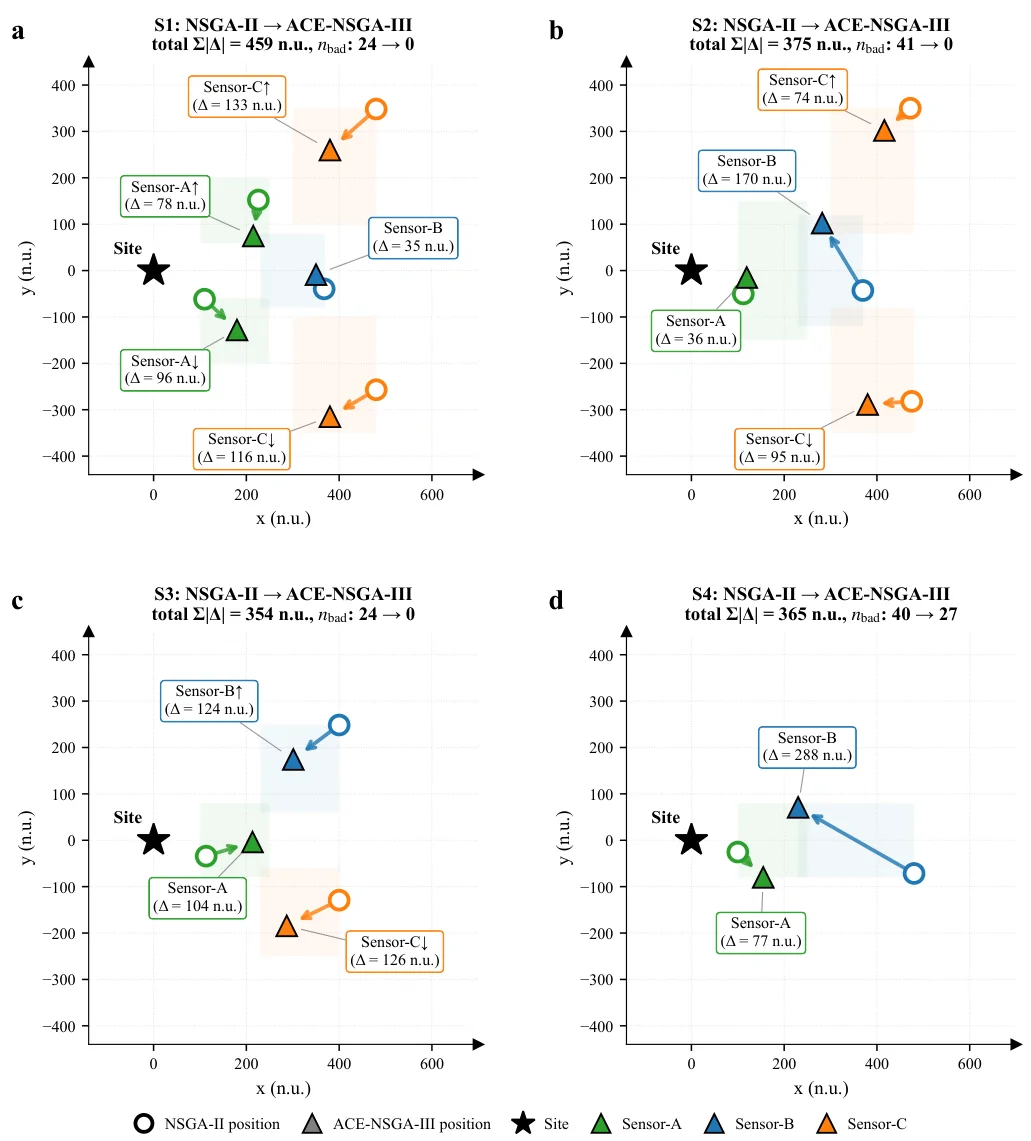
Sensor migration from the NSGA-II deployment (open circles) to the ACE-NSGA-III deployment (filled triangles) on four scenarios: (**a**) S1; (**b**) S2; (**c**) S3; (**d**) S4. Each label gives the sensor and its displacement Δ, and ↑ or ↓ marks the sensor whose placement box (shaded) lies above or below the centerline; the panel title gives the total displacement and $n_{\mathrm{bad}}$ of the two deployments. Total migration ranges from 354 to 459 n.u.; on S4, Sensor-B moves 288 n.u. from (480,−72) to (230,+71).

**Figure A4:**
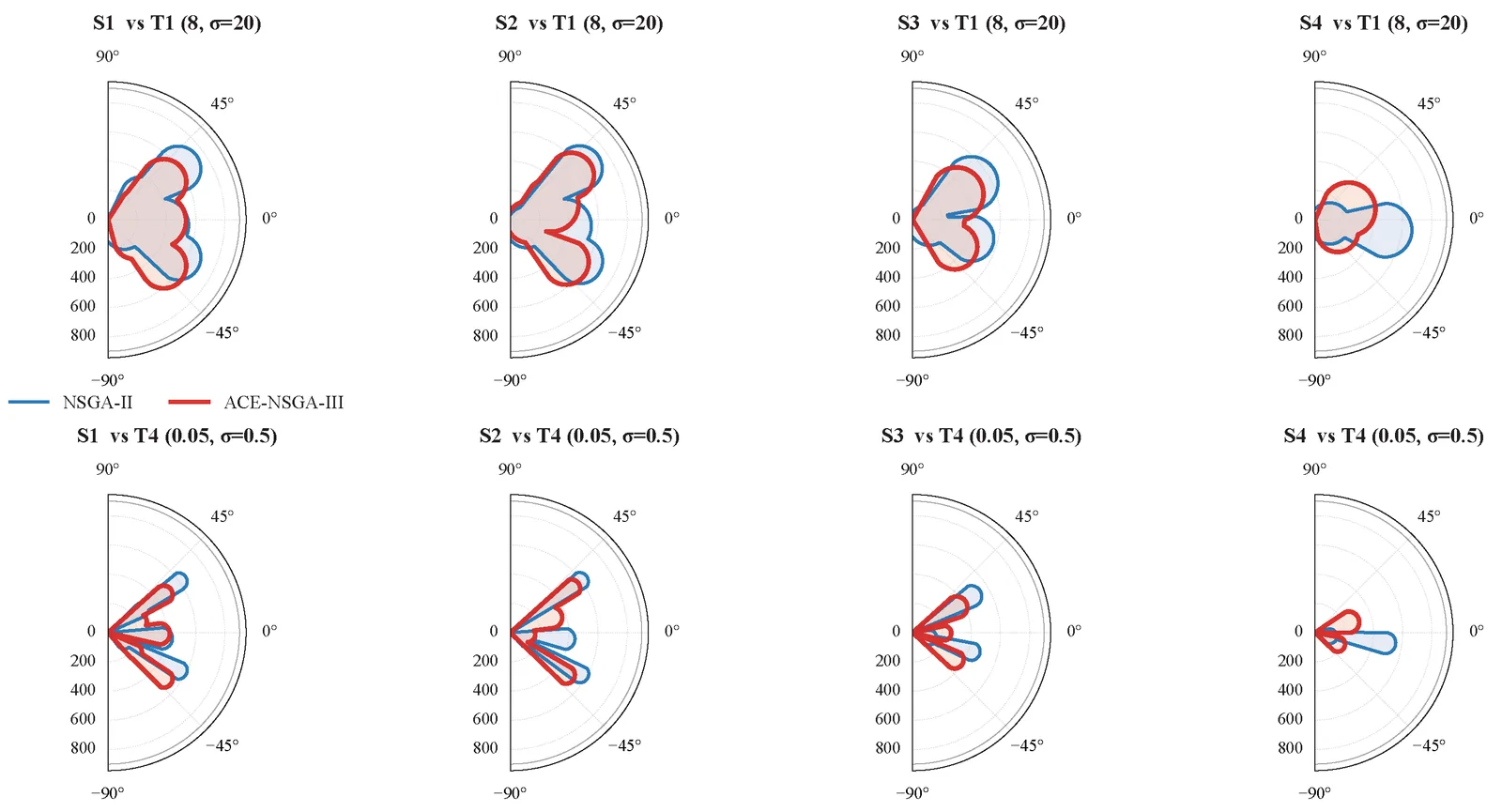
Angular coverage (maximum detection radius per approach angle) of NSGA-II (blue) and ACE-NSGA-III (red) on four scenarios, against a T1 target (**top row**) and a T4 target (**bottom row**). The outer arc marks the 900 n.u. start radius; the radius shrinks by a factor of about 4 from T1 to T4.

**Figure A5:**
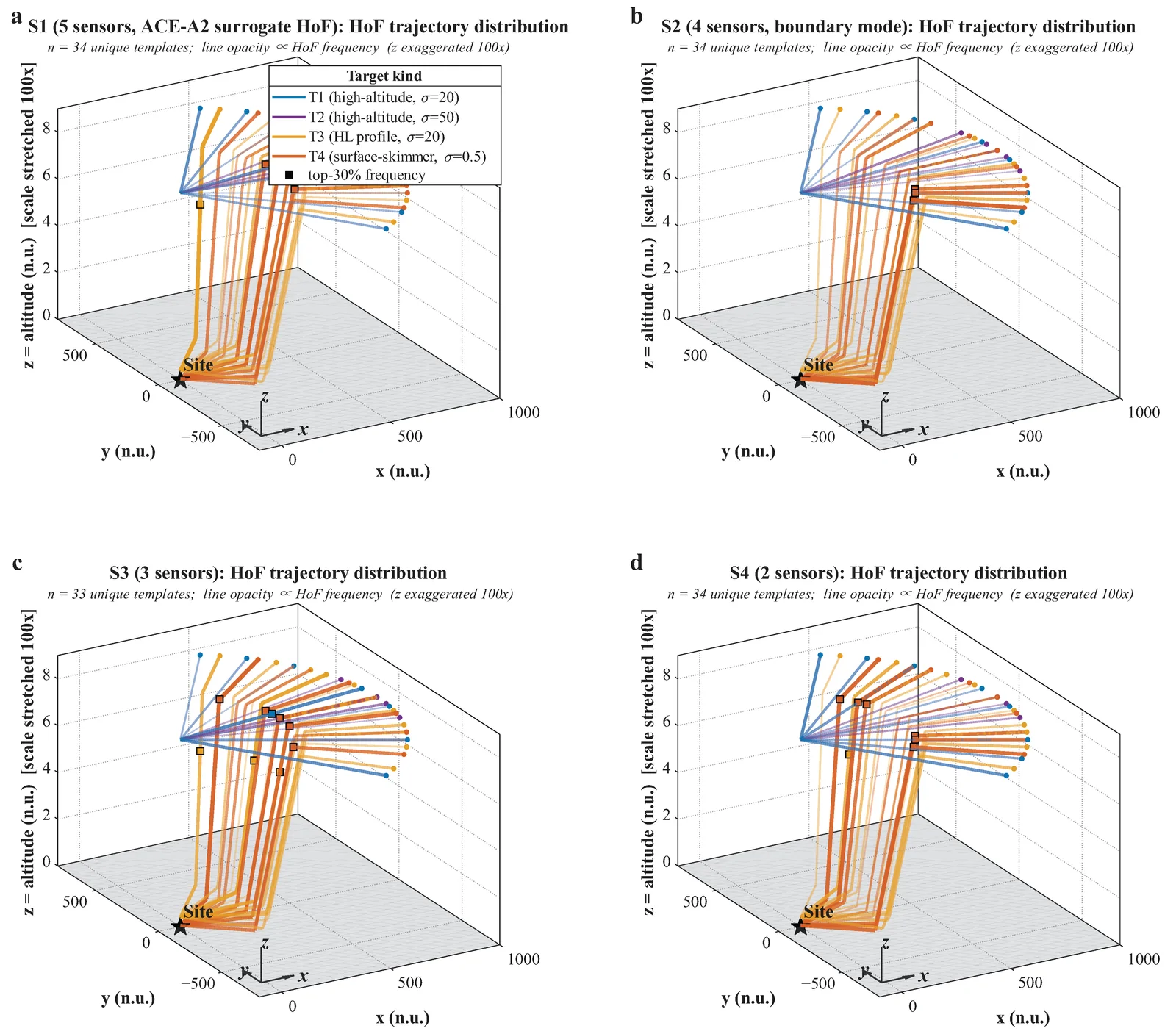
Hall-of-fame cases over 5 seeds on the four scenarios, (**a**) S1, (**b**) S2, (**c**) S3 and (**d**) S4, drawn as HL profile trajectories with opacity proportional to how often a template appears in the HoF and color by kind; filled squares mark the top 30% templates. On S1, where ACE-A6 runs CCMO and keeps no HoF, the HoF of an ACE-A2 run is shown. The vertical axis is stretched 100×.

**Deployment geometry.** Figure 9 shows how the training regime changes the deployment. Under single-case training, NSGA-II places the sensors near the centerline, which is reasonable for a symmetric case but leaves flank sectors open. Under co-evolutionary training, the S3 deployment separates Sensor-B (y=+174 n.u.) and Sensor-C (y=−185 n.u.), and the S4 deployment moves Sensor-B by 288 n.u., to 71 n.u. above the centerline, a shift that the single training case gives no reason to make (Appendix B, Figure A3). Figure A4 (Appendix B) compares the angular coverage of the two training regimes against T1 and T4 targets. The hall of fame records the compositions that keep defeating the deployment. On S3, it holds a mixed set of kinds at flank angles, and on S4, it concentrates on low-altitude, low-cross-section templates in narrow sectors (Appendix B, Figure A5). This list is an output of the method that an operator can read directly as the traffic to which the deployment is most exposed.

## 7. Discussion

**Where the improvement comes from.** The physical origin of the per-kind failures is the horizon collapse of the low-mounted sensor. For T3, the effective radius of Sensor-A drops from 141 n.u. (cross-section-limited at 8 n.u. altitude) to 104 n.u. (horizon-limited at 0.4 n.u.), and for T4, the cross-section-limited radius of 56 n.u. binds at every altitude. A deployment trained on one case can leave the sector through which such targets arrive uncovered, and on a test case that concentrates them there, a whole kind is missed. The experiments of Section 6.3 show that the remedy is the training framework, which exposes the deployment during training to several kind-diverse compositions that include the hard ones. Section 6.4 shows that the fairness objective turns that exposure into lower per-kind miss rates. ACE-A6 is an instance that reaches these levels at a budget comparable to that of the baselines, with the largest margin where the network is smallest.

**Sensor-count dispatch.** ACE-A6 and its predecessor ACE-A5 differ only in the dispatch. ACE-A5 sends every network with four or more sensors to Easy mode and decides the other modes by a three-generation probe, whereas ACE-A6 gives four-sensor and two-sensor networks the dual-branch ensemble. On S2, this is the difference between $f_{4,\max}=1$ (ACE-A5, which runs CCMO there) and 0 (ACE-A6). Section 6.5 shows the limit of the static rule. The rule is a proxy for coverage capacity and is not always the best choice, although its choices cost little on the held-out scenarios.

**Computational cost.** One (deployment, case) evaluation takes about 0.5 ms, so the budgets of Table 3 correspond to between about 1.5 and 6 s of evaluation time per run on one processor core, and a 30-seed scenario takes minutes.

**Limitations and scope.** The evidence is entirely simulation-based, and the sensing model is first order. Section 6.6 re-introduces five non-ideal effects by re-evaluating deployments, but the deployments are not optimized under them, and multipath, interference and site-specific terrain are not modeled. The physical calibration of the normalized unit is not released, whereas the complete normalized parameter set is given in Table 2 and Table A1. The deployment is a static 2D placement computed offline, with fixed sensor altitudes. The conclusions hold within the target library. Shifts that leave it, such as a wider approach sector or an unseen kind, defeat every method tested, so the library must be built to cover the expected traffic.

## 8. Conclusions

This paper formulates sensor deployment for layered low-altitude airspace monitoring against worst-case traffic composition, with an HL-profile target model, a 35-template library in four kinds and a per-kind fairness objective $f_{4}$, and proposes the ACE training framework with its instance ACE-A6.

Against four state-of-the-art optimizers in their published single-case form, ACE-A6 lowers the worst per-kind miss rate on the four-, three- and two-sensor scenarios from 1 to 0, 0 and 0.5, with large effect sizes on two independent test sets. It also lowers the median number of test cases with a missed target from 41, 24 and 40–55 to 0, 0 and 27. The equal-budget experiments locate this gain in the training framework. Each of five optimizers placed inside ACE also breaks the $f_{4,\max}=1$ ceiling, and ACE-A6 performs on par with them, with its clearest margin on the two-sensor scenario. The fairness objective is effective only together with kind-diverse training cases. The advantage over the published baselines holds under probabilistic detection, masking, environmental attenuation, position error, M-of-N confirmation with false alarms and moderate library shifts, and it disappears when the test traffic leaves the training library.

Future work includes optimizing deployment under the stochastic sensing model rather than only evaluating it there, adding sensor altitude as a decision variable and a dispatch rule based on coverage capacity rather than sensor count, and implementing online relocation of sensors from observed tracks.

## Figures and Tables

**Table 1:** Line-of-sight (LoS) horizon $R_{\mathrm{hor}}$ in normalized simulation units (n.u.) for each sensor type against high and low altitude targets. Low-altitude flight imposes a 39–73% horizon reduction.

Sensor	$h_{s}$ (n.u.)	$h_{t}=8$	$h_{t}=0.4$	Reduction
Sensor-A	0.028	390.3	104.2	−73%
Sensor-B	8	737.2	451.0	−39%
Sensor-C	5	660.6	374.7	−43%

**Table 2:** Complete parameter set of the simulation model. Lengths and altitudes in n.u., speeds in n.u./h, times in s and cross sections in the unit of $\sigma_{\mathrm{ref}}$. The placement boxes and ordering constraints of every scenario are listed in Table A1.

Quantity	Value
Horizon constant κ; reference cross section $\sigma_{\mathrm{ref}}$	130.3; 5
Sensor-A/Sensor-B/Sensor-C: height $h_{s}$	0.028/8/5
Sensor-A/Sensor-B/Sensor-C: reference range $R_{\mathrm{ref}}$	100/130/110
Target start radius $R_{\mathrm{start}}$; near-field exclusion radius	900; 5
Time step; horizon $T_{\mathrm{sim}}$; second-wave launch delay	30; 4500; 300
Confirmation rule	3 consecutive steps in range of ≥1 sensor
T1: altitude, speed, σ	8, 800, 20 (constant altitude)
T2: altitude, speed, σ	8, 600, 50 (constant altitude)
T3: $h_{\mathrm{high}}\to h_{\mathrm{low}}$, $r_{\mathrm{dive}}$, $r_{\mathrm{skim}}$, speed, σ	8→0.4, 500, 350, 800, 20
T4: $h_{\mathrm{high}}\to h_{\mathrm{low}}$, $r_{\mathrm{dive}}$, $r_{\mathrm{skim}}$, speed, σ	8→0.05, 450, 300, 1000, 0.5
Library: templates per kind (T1/T2/T3/T4)	8/5/12/10, angles in $\left[-55^{\circ },55^{\circ }\right]$
Score weights in $F=w_{1}f_{1}+w_{2}f_{2}+w_{3}f_{3}$	0.4, 0.4, 0.2

**Table 3:** Training evaluation budget, counted as (deployment, case) evaluations by instrumentation; median over 30 seeds. ACE-A6 range over seeds in brackets. RS, random search; NSGA-II, non-dominated sorting genetic algorithm II; NSGA-III(4obj), NSGA-III with four objectives; PW-NSGA-II, priority-weighted NSGA-II; C-TAEA, constrained two-archive evolutionary algorithm; MaOEA-PDE, many-objective evolutionary algorithm with Pareto front density estimation; EnMOGKSO, enhanced multi-objective Genghis Khan shark optimizer.

Method	S1	S2	S3	S4
RS	5760	5760	5760	5760
NSGA-II	5800	5800	5800	5800
C-TAEA	5800	5800	5800	5800
MaOEA-PDE	5800	5800	5800	5800
EnMOGKSO	5800	5800	5800	5800
NSGA-III(4obj)	5220	5220	5220	5220
PW-NSGA-II	9575	9305	7861	6938
CCMO	11,600	11,600	11,600	11,600
**ACE-A6**	11,601	7866	2725	7225
	(11,601)	[3548, 9961]	[2371, 5490]	[4523, 10,176]

**Table 4:** Median (over 30 seeds) test set metrics on the primary test set (60 held-out cases). ^⋆^ marks the best per (scenario, metric); lower is better. All ACE variants are reported in Table A2.

Method	S1 (5 Sensors)		S2 (4 Sensors)		S3 (3 Sensors)		S4 (2 Sensors)	
	$F_{\max}$	$f_{4,\max}$	$F_{\max}$	$f_{4,\max}$	$F_{\max}$	$f_{4,\max}$	$F_{\max}$	$f_{4,\max}$
RS	0.611	1.000	0.738	1.000	0.751	1.000	1.098	1.000
NSGA-II	0.536	**0.000 ^⋆^**	0.765	1.000	0.678	1.000	0.957	1.000
PW-NSGA-II	0.523	0.000	0.767	1.000	0.931	1.000	0.955	1.000
NSGA-III(4obj)	0.541	0.000	0.766	1.000	0.675	1.000	1.014	1.000
CCMO	**0.522 ^⋆^**	0.000	0.765	1.000	0.685	1.000	1.015	1.000
C-TAEA	0.563	0.000	0.764	1.000	0.684	1.000	0.956	1.000
MaOEA-PDE	0.564	0.250	0.765	1.000	0.681	1.000	1.016	1.000
EnMOGKSO	0.578	0.250	0.765	1.000	0.690	1.000	1.016	1.000
**ACE-A6**	**0.522 ^⋆^**	**0.000 ^⋆^**	**0.620 ^⋆^**	**0.000 ^⋆^**	**0.652 ^⋆^**	**0.000 ^⋆^**	**0.844 ^⋆^**	**0.500 ^⋆^**

**Table 5:** Pairwise Wilcoxon test of ACE-A6 against each external method on $f_{4,\max}$ for S2 and S3 (30 seeds, primary test set). $p_{\mathrm{Bonf}}$ is Bonferroni-corrected over K=180. Sig.: **, $p_{\mathrm{Bonf}}<10^{-2}$; ***, $p_{\mathrm{Bonf}}<10^{-3}$.

Baseline	Median	δ	$p_{\mathrm{Bonf}}$	Sig.
**Scenario S2 (**$N_{s}=4$, D=8**)**				
RS	1.000	+0.75	$2.5\times 10^{-3}$	**
NSGA-II	1.000	+0.80	$2.9\times 10^{-3}$	**
PW-NSGA-II	1.000	+0.80	$1.3\times 10^{-3}$	**
NSGA-III(4obj)	1.000	+0.82	$1.2\times 10^{-3}$	**
CCMO	1.000	+0.83	$1.1\times 10^{-3}$	**
C-TAEA	1.000	+0.79	$1.9\times 10^{-3}$	**
MaOEA-PDE	1.000	+0.82	$1.2\times 10^{-3}$	**
EnMOGKSO	1.000	+0.76	$2.8\times 10^{-3}$	**
ACE-A6	**0.000**	–	–	–
**Scenario S3 (**$N_{s}=3$, D=6**)**				
RS	1.000	+0.71	$7.5\times 10^{-3}$	**
NSGA-II	1.000	+0.72	$3.9\times 10^{-3}$	**
PW-NSGA-II	1.000	+0.80	$1.4\times 10^{-3}$	**
NSGA-III(4obj)	1.000	+0.83	$9.8\times 10^{-4}$	***
CCMO	1.000	+0.87	$7.6\times 10^{-4}$	***
C-TAEA	1.000	+0.67	$5.5\times 10^{-3}$	**
MaOEA-PDE	1.000	+0.83	$8.9\times 10^{-4}$	***
EnMOGKSO	1.000	+0.85	$9.0\times 10^{-4}$	***
ACE-A6	**0.000**	–	–	–

**Table 6:** Same-framework comparison (primary test set, 30 seeds). Each row gives, for one training regime, the range of the median $F_{\max}$ over the five optimizers, the number of optimizers with median $f_{4,\max}=0$ and the number of the 15 comparisons with ACE-A6 (five optimizers by $F_{\max}$, $f_{4,\max}$ and $n_{\mathrm{bad}}$) in which ACE-A6 is significantly better or worse (Holm, p<0.05); in the columns A6 ±, the first number counts the comparisons in which ACE-A6 is better and the second those in which it is worse.

Regime	S2 (4 Sensors)			S3 (3 Sensors)			S4 (2 Sensors)		
	$F_{\max}$	$f_{4}=0$	A6 ±	$F_{\max}$	$f_{4}=0$	A6 ±	$F_{\max}$	$f_{4}=0$	A6 ±
Native, single case	0.764–0.765	0/5	15/0	0.678–0.690	0/5	8/0	0.956–1.016	0/5	15/0
Static, A6 budget	0.580–0.639	4/5	0/0	0.657–0.700	2/5	0/0	0.865–0.883	0/5	0/0
Co-evolved, A6 budget	0.585–0.641	1/5	0/0	0.651–0.709	1/5	0/0	0.873–0.954	0/5	0/0
Static, 11,600	0.571–0.603	5/5	0/1	0.617–0.636	5/5	0/1	0.822–0.867	0/5	0/0
Co-evolved, 11,600	0.586–0.647	4/5	0/0	0.625–0.686	4/5	0/0	0.825–0.935	0/5	0/0
ACE-A6	0.620, $f_{4,\max}=0$			0.652, $f_{4,\max}=0$			0.844, $f_{4,\max}=0.5$		

**Table 7:** Factorial on objective set and training cases (NSGA-III, 5800 evaluations, 30 seeds, primary test set). Entries are the median $f_{4,\max}$, with the median $F_{\max}$ in parentheses.

Scenario	Single Case		Static Multi-Case		Co-Evolved	
	With $f_{4}$	Without	With $f_{4}$	Without	With $f_{4}$	Without
S1	1.00 (0.588)	1.00 (0.588)	0.00 (0.542)	0.00 (0.541)	0.00 (0.566)	0.00 (0.560)
S2	1.00 (0.765)	1.00 (0.765)	0.00 (0.593)	0.50 (0.648)	0.00 (0.603)	0.50 (0.662)
S3	1.00 (0.687)	1.00 (0.677)	0.00 (0.634)	0.50 (0.708)	0.42 (0.679)	0.00 (0.651)
S4	1.00 (0.957)	1.00 (1.017)	0.67 (0.883)	0.88 (0.925)	0.67 (0.870)	1.00 (0.917)

**Table 8:** Dispatch rule on ten held-out scenarios (30 seeds; test cases drawn from the library of the scenario). The columns give the median $F_{\max}$/$f_{4,\max}$ of each mode, the mode chosen by the rule, the oracle mode, the regret of the rule in $F_{\max}+0.5f_{4,\max}$ and the Holm-corrected *p* of rule versus oracle. In the Scenario column, 2A+1B+1C denotes two Sensor-A, one Sensor-B and one Sensor-C; Dual is the dual-branch ensemble shared by the Boundary and Hard modes.

	Scenario	Easy	Medium	Dual	Rule	Oracle	Regret	$p_{\mathrm{Holm}}$
G1	$N_{s}=4$, 2A+1B+1C	0.668/1.00	0.674/0.00	0.633/0.00	Dual	Dual	0	1
G2	$N_{s}=3$, 2A+1B	0.736/1.00	0.692/0.00	0.681/0.00	Medium	Dual	0.012	1
G3	$N_{s}=3$, 1A+2C	0.784/1.00	0.734/0.50	0.663/0.00	Medium	Dual	0.322	0.062
G4	$N_{s}=2$, 1B+1C	1.028/1.00	0.992/1.00	0.974/1.00	Dual	Dual	0	1
G5	$N_{s}=6$, 2A+2B+2C	0.516/0.00	0.539/0.00	0.527/0.00	Easy	Easy	0	1
G6	$N_{s}=1$, 1B	1.154/1.00	0.995/1.00	0.994/1.00	Dual	Dual	0	1
G7	$N_{s}=3$, S3 sensors, region ×0.7	0.625/0.00	0.648/0.00	0.641/0.00	Medium	Easy	0.022	1
G8	$N_{s}=4$, S2 sensors, sector $\pm 80^{\circ }$	0.910/1.00	0.958/1.00	0.956/1.00	Dual	Easy	0.011	1
G9	$N_{s}=3$, S3 sensors, lower HL, −3 dB	0.833/1.00	0.778/0.50	0.774/0.50	Medium	Dual	0.055	1
G10	$N_{s}=2$, S4 sensors, sector $\pm 80^{\circ }$	1.230/1.00	1.152/1.00	1.158/1.00	Dual	Medium	0.006	1

**Table 9:** ACE family progression, S3 median over 30 seeds (primary test set).

Variant	$F_{\max}$	$f_{4,\max}$	Key Feature
ACE-NSGA-III	0.725	0.50	4 objectives + co-evolution
ACE-A	0.696	0.50	+ two-phase schedule
ACE-A-tuned	0.685	0.50	+ tuned hyperparameters
ACE-A2	0.676	0.50	+ clustered HoF + dual archive
ACE-A3	0.664	0.00	+ relative-growth plateau
ACE-A4	0.671	0.17	running-maximum patience
ACE-A5	0.657	0.00	+ mode dispatch with probe
**ACE-A6**	**0.652**	**0.00**	+ static sensor-count dispatch

## Data Availability

The ACE-A6 reference implementation, simulation model, 30-seed result archive, scripts of the experiments added in this revision, and figure-generation scripts are available from the corresponding author upon reasonable request.

## Abbreviations and Nomenclature

The following abbreviations and symbols are used in this manuscript:

ACE	Adaptive co-evolution (this work)
HL	High–low transition (trajectory profile)
LoS	Line of sight
HoF	Hall of Fame
RCS	Target cross-section (scattering signature, σ)
n.u.	Normalized simulation unit
SBX	Simulated binary crossover
RNG	Random number generator
UAV	Unmanned aerial vehicle
RS	Random search
NSGA-II	Non-dominated sorting genetic algorithm II
PW-NSGA-II	Priority-weighted NSGA-II
NSGA-III	Reference-point-based successor of NSGA-II
CCMO	Coevolutionary constrained multiobjective optimization
C-TAEA	Constrained two-archive evolutionary algorithm
MaOEA-PDE	Many-objective EA with Pareto-density estimation
EnMOGKSO	Enhanced multi-objective Genghis Khan shark optimizer
M, |M|=35	Target library and its size
$a\in M^{12}$	A case: 12 templates from the library
$x\in R^{D}$	Deployment decision vector ($D=2N_{s}$)
$N_{s}$	Sensor count (5/4/3/2 for S1/S2/S3/S4)
$f_{1},f_{2},f_{3}$	Detection range, time and track-duration objectives
$f_{4}=\max_{k}{\mathrm{MR}}_{k}$	Worst per-kind miss rate
*F*	Scalar score $0.4f_{1}+0.4f_{2}+0.2f_{3}$
$F_{\max},f_{4,\max}$	Largest *F* and $f_{4}$ over the test cases
$n_{\mathrm{bad}}$, $n_{\mathrm{cat}}$	Test cases with a missed target ($f_{4}>0$)/a missed kind ($f_{4}=1$)
$R_{\mathrm{hor}},R_{\mathrm{RCS}},R_{\mathrm{eff}}$	Horizon-limited, cross-section-limited, effective range (n.u.)
$h_{s},h_{t},\sigma_{t}$	Sensor altitude, target altitude, target cross-section
D,A,HoF	Deployment, case, hall-of-fame populations
$n_{d},n_{a},k_{\mathrm{hof}}$	Deployment population, case population, HoF size
δ	Cliff’s delta effect size

## Appendix A. Scenario Definitions and Full Results

**Table A1:** Placement boxes (*x* and *y* ranges in n.u.) and ordering constraints of the four scenarios. Ordering constraints: every Sensor-B lies at least 60 n.u. further from the site in *x* than every Sensor-A, and every Sensor-C at least 30 n.u. further than Sensor-B (S1, S2); in S3, Sensor-B and Sensor-C lie at least 60 n.u. beyond Sensor-A; in S4, Sensor-B lies at least 60 n.u. beyond Sensor-A.

Scenario	Sensors and Placement Boxes
S1	A: x∈[100,250], y∈[60,200] and y∈[−200,−60]; B: [230,370]×[−80,80];
	C: x∈[300,480], y∈[100,350] and y∈[−350,−100]
S2	A: [100,250]×[−150,150]; B: [230,370]×[−120,120];
	C: x∈[300,480], y∈[80,350] and y∈[−350,−80]
S3	A: [100,250]×[−80,80]; B: [230,400]×[60,250]; C: [230,400]×[−250,−60]
S4	A: [100,250]×[−80,80]; B: [230,480]×[−80,80]

**Table A2:** Median (over 30 seeds) test set metrics of all 16 methods on the primary test set; this is the main results table of the original submission. ^⋆^ marks the best per (scenario, metric).

Method	S1		S2		S3		S4	
	$F_{\max}$	$f_{4,\max}$	$F_{\max}$	$f_{4,\max}$	$F_{\max}$	$f_{4,\max}$	$F_{\max}$	$f_{4,\max}$
RS	0.611	1.000	0.738	1.000	0.751	1.000	1.098	1.000
NSGA-II	0.536	**0.000 ^⋆^**	0.765	1.000	0.678	1.000	0.957	1.000
PW-NSGA-II	0.523	0.000	0.767	1.000	0.931	1.000	0.955	1.000
NSGA-III(4obj)	0.541	0.000	0.766	1.000	0.675	1.000	1.014	1.000
ACE-NSGA-III	0.580	0.000	0.689	0.500	0.725	0.500	0.960	1.000
ACE-A	0.574	0.000	0.659	0.500	0.696	0.500	0.956	1.000
ACE-A2	0.566	0.000	0.624	0.167	0.676	0.500	0.934	0.708
ACE-A3	0.586	0.000	0.657	0.500	0.664	0.000	0.927	1.000
ACE-A5	**0.522**	0.000	0.765	1.000	0.657	0.000	0.934	0.708
**ACE-A6**	**0.522 ^⋆^**	**0.000 ^⋆^**	**0.620 ^⋆^**	**0.000 ^⋆^**	**0.652 ^⋆^**	**0.000 ^⋆^**	**0.844 ^⋆^**	**0.500 ^⋆^**
CCMO	**0.522 ^⋆^**	0.000	0.765	1.000	0.685	1.000	1.015	1.000
C-TAEA	0.563	0.000	0.764	1.000	0.684	1.000	0.956	1.000
MaOEA-PDE	0.564	0.250	0.765	1.000	0.681	1.000	1.016	1.000
EnMOGKSO	0.578	0.250	0.765	1.000	0.690	1.000	1.016	1.000

## Appendix C. A Dispatch-Free Variant (ACE-A7)

The observations of Section 6.3, that a fixed set of kind-diverse cases is already effective and that co-evolution is noisy at small budgets, motivate a dispatch-free variant. ACE-A7 trains one NSGA-II-type population for every scenario on six fixed kind-diverse anchor cases together with four co-evolved cases and a clustered HoF of four, uses the conditional value at risk of *F* over the worst 30% of the cases as an objective, and caches (deployment, case) evaluations. Because its design draws on results obtained on the primary test set, its design and acceptance criterion (significantly better than every same-framework arm on $F_{\max}$ or $f_{4,\max}$ on at least three scenarios) are fixed in writing before the first run, and it is judged on the independent test set.

At the ACE-A6 budget, ACE-A7 meets the criterion on none of the four scenarios. Its median $F_{\max}$ on the independent test set is 0.529, 0.573, 0.661 and 0.828 on S1–S4 (ACE-A6: 0.513, 0.605, 0.646, 0.808). On the held-out scenarios of Table 8, at the budget spent by the mode the rule selects, its median $F_{\max}$ is lower than that of the mode chosen by the rule on nine of the ten (descriptive, not tested), including G3 (0.656 against 0.734 for the Medium mode). Training on fixed kind-diverse anchors is therefore a plausible way to remove the dispatcher, but it does not improve on ACE-A6 on the scenarios ACE-A6 is designed for, and it is not proposed as a replacement.
